# Progressive Design of a Ranatuerin-2 Peptide from *Amolops wuyiensis*: Enhancement of Bioactivity and In Vivo Efficacy

**DOI:** 10.3390/antibiotics13010005

**Published:** 2023-12-19

**Authors:** Aifang Yao, Tianxing Liu, Yuhai Cai, Siqi Zhou, Xiaoling Chen, Mei Zhou, Chengbang Ma, Tianbao Chen, Chris Shaw, Lei Wang

**Affiliations:** 1 College of Biological Science and Engineering, Fuzhou University, Fuzhou 350108, China; 2 School of Pharmacy, Queen’s University Belfast, 97 Lisburn Road, Belfast BT9 7BL, UK; tliu18@qub.ac.uk (T.L.); ycai15@qub.ac.uk (Y.C.); szhou13@qub.ac.uk (S.Z.); m.zhou@qub.ac.uk (M.Z.); c.ma@qub.ac.uk (C.M.); t.chen@qub.ac.uk (T.C.); chris.shaw@qub.ac.uk (C.S.); l.wang@qub.ac.uk (L.W.)

**Keywords:** antimicrobial peptides (AMPs), peptide design, multidrug-resistant bacteria, ranatuerin, drug optimisation

## Abstract

Antimicrobial peptides (AMPs) that exert multiple functions are considered promising candidates to combat the bacterial drug resistance crisis. Nowadays, targeted peptide modification has been widely recognised to improve biological activity and make up for deficiencies in clinical applications such as toxicity. In this study, a helix-loop peptide was isolated and identified from the skin secretion of the Wuyi torrent frog *Amolops wuyiensis*, namely, ranatuerin-2-AW (R2AW) (GFMDTAKNVAKNVAATLLDKLKCKITGGC). Target modifications were made to R2AW to study the structure–activity relationships and to optimise its bioactivities. Five analogues were progressively designed via residue substitution and truncation and the antibacterial and anticancer activities were evaluated. We found that the serine-substitution and cyclic-domain-deletion products showed similar antibacterial activity to the natural peptide R2AW, implying that the disulphide bridge and Rana box were dispensable for the antibacterial activity of ranatuerin-2 peptides. Notably, the cationicity- and hydrophobicity-enhanced variant, [Lys^4,19^, Leu^20^]R2AW(1-22)-NH_2_, exhibited significantly optimised antibacterial and anticancer activities. Additionally, it killed bacteria by membrane disruption at a highly efficient rate. Moreover, [Lys^4,19^, Leu^20^]R2AW(1-22)-NH_2_ exerted potential in vivo efficacy in a methicillin-resistant *Staphylococcus aureus* (MRSA)-infected waxworm model. Overall, this study demonstrated some rational design ideas for optimising the dual antibacterial and anticancer activities of ranatuerin-2 peptides and it proposes [Lys^4,19^, Leu^20^]R2AW(1-22)-NH_2_ as an appealing candidate for therapeutic development.

## 1. Introduction

The high mortality rates and economic burden caused by drug-resistant pathogens are considered among the most serious health threats worldwide [1,2]. Recent statistics have revealed that infection mortality is on an increasing trend with limited novel therapies available [3]. Another challenge is the increasing incidence of cancer. According to one report, there were approximately 18.3 million new cancer cases globally in 2018, with lung, breast, prostate, and colorectal cancer accounting for most diagnosed cases [4,5]. Moreover, researchers have pointed out that chronic infections are related to cancer [6,7,8]. Currently, commercially available drugs for the treatment of infection and cancer have low selectivity and have induced severe drug resistance. Therefore, it is urgent to discover new agents that can kill or inhibit the growth of different microorganisms and cancer cells.

Accordingly, the structures of more than 3000 antimicrobial peptides (AMPs) are currently archived in the antimicrobial peptide database (APD3) [9]. AMPs from amphibian skin secretions are generally characterised by having cationic and amphipathic properties and may play important roles in host defence systems. As stated, AMPs possess antibacterial and antifungal activities and some also have shown significant anticancer activities [9,10]. In general, the membrane-targeting mechanism of AMPs can be explained by models, such as pore and carpet models. The advantages of rapid killing mechanisms and less possibility of drug resistance induction make AMPs a most promising candidate group for evaluation to solve the crisis [11]. 

AMPs from Ranidae frogs have significant structural diversity and have been divided into several families, including brevinins and ranatuerins [12]. Ranatuerin-2 was first isolated and identified from the skin secretion of *Lithobates catesbeianus* and is commonly found in North American frogs and the Chinese bamboo leaf odorous frog [13,14]. The primary structures of ranatuerin-2 peptides are poorly conserved, characterised by several residue deletions and two invariant cysteines forming the cyclic hexapeptide or heptapeptide domain (Rana box) at the C-terminus [15]. In general, peptides from the ranatuerin-2 family show a broad-spectrum activity against bacteria with low haemolysis [12]. As well as this, peptides such as ranatuerin-2PLx can prevent cancer cell proliferation, indicating the dual therapeutic potential of these peptides [16]. Hence, this small peptide with low toxicity and lack of drug resistance has excellent potential and value to be developed into a dual antibacterial and anticancer agent.

Generally, the comprehensive study of structure–activity relationships will contribute to the rational design of AMPs as a substitute for traditional agents for clinical applications. So far, the role of the distinct cyclic domain, the Rana box, in helix-loop AMPs remains ambiguous. As reported, the C-terminal heptapeptide domain is crucial for maintaining the bioactivities for B1CTcu5, whereas it is dispensable for nigrocin-HL [17,18]. However, the functions of the Rana box and the disulphide bridge in ranatuerin-2 peptides have not been clarified clearly. 

In this study, a ranatuerin-2 peptide (R2AW) was isolated and identified from the skin secretion of *Amolops wuyiensis* and the roles of the cyclic heptapeptide domain and the cysteine bridge in the peptide were studied. Apart from the study of the structure–activity relationships, this work focused on specific modifications of peptides to optimise their antibacterial and anticancer activities. Hence, a series of peptides were chemically synthesised by use of a solid-phase peptide synthesiser. After that, reverse-phase high-performance liquid chromatography (RP-HPLC) and matrix-assisted laser desorption/ionisation time-of-flight (MALDI-TOF) mass spectrometry were applied for peptide purification and identification. The in vitro antibacterial and antibiofilm abilities were evaluated using different bacterial strains. The antiproliferative effect was assayed on human cancer cell lines. Additionally, the haemolytic effect was assessed on horse red blood cells. Moreover, the in vivo antibacterial efficacy was examined using a methicillin-resistant *Staphylococcus aureus* (MRSA)-infected larvae model.

## 2. Results

### 2.1. “Shotgun” Cloning of R2AW from Amolops Wuyiensis Skin Secretion-Derived cDNA Library

**Figure 1:**
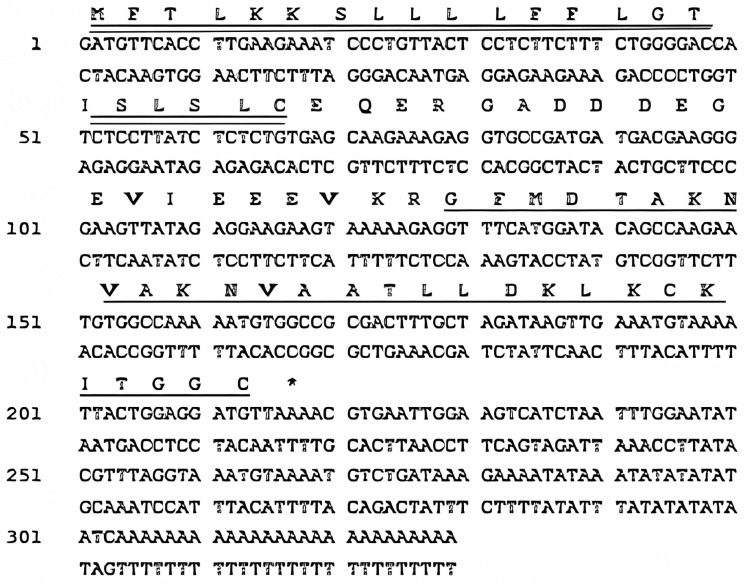
Nucleotide sequence of cDNA encoding *Amolops wuyiensis* antimicrobial peptide (ranatuerin-2-AW) and translated open-reading frame (ORF) amino acid sequence of the biosynthetic precursor. The putative signal peptide (double-underlined), mature peptide (single-underlined), and stop codon (asterisk) are indicated.

The cDNA of the novel peptide, ranatuerin-2-AW (R2AW), was consistently cloned from the skin secretion of *Amolops wuyiensis*. The obtained nucleotide sequence is shown in Figure 1. The sequence was deposited in the GenBank with the accession number HF912236. The N-terminal 22 amino acid residues encoded a putative signal peptide, followed by an acidic spacer peptide domain of 17 amino acids. After a basic –KR- pro-peptide convertase cleavage site, a mature peptide domain of 29 amino acid residues was located at the C-terminus. 

**Figure 2:**
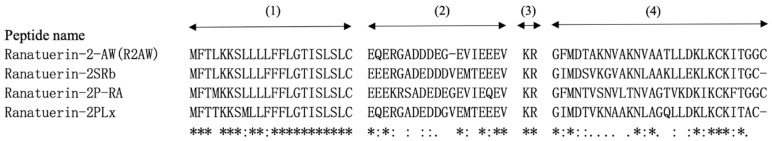
Sequence alignment of R2AW and ranatuerin family peptide precursors derived from different frog species. The domains are divided into four parts, (**1**) the putative signal peptide, (**2**) the acidic spacer region, (**3**) the convertase processing site, and (**4**) the mature peptide. An asterisk (*) indicates that the residue in this position is fully conserved in the sequence alignment. A colon (:) indicates conservation between groups with strongly similar properties, while a period (.) indicates conservation between groups with weakly similar properties.

Additionally, the sequences of R2AW, ranatuerin-2SRb [19], ranatuerin-2P-RA, and ranatuerin-2PLx [16] were aligned using the Uniprot database (available online: https://www.uniprot.org/ (accessed on 9 March 2023)), as shown in Figure 2. The mature peptide domain of R2AW was a highly conserved ranatuerin-2-related peptide.

### 2.2. Peptide Design

**Figure 3:**
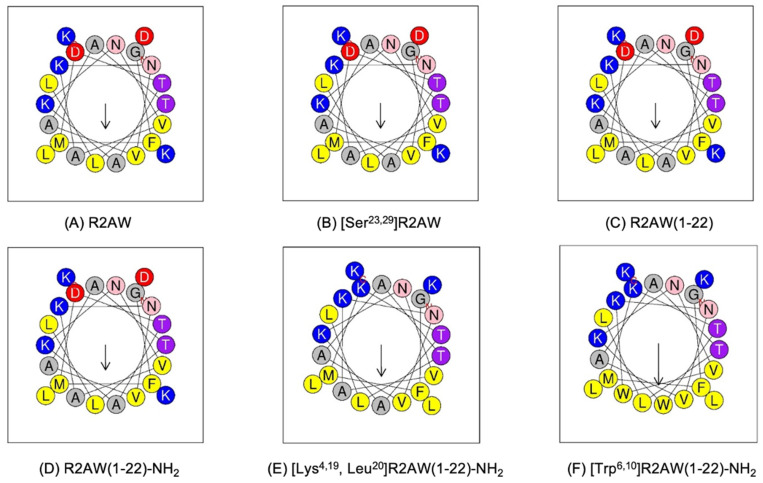
Helical wheel plots of (**A**) R2AW, (**B**) [Ser^23,29^]R2AW, (**C**) R2AW(1-22), (**D**) R2AW(1-22)-NH_2_, (**E**) [Lys^4,19^, Leu^20^]R2AW(1-22)-NH_2_, and (**F**) [Trp^6,10^]R2AW(1-22)-NH_2_. The arrow points from the hydrophilic side to the hydrophobic side. Residues are colour-coded with hydrophobic residues in yellow, basic residues in dark blue, acidic residues in red, asparagine in pink, threonine in purple, and alanine and glycine in grey circles.

Herein, we have proposed a progressive peptide design idea, and the physicochemical parameters of the peptides were evaluated by Heli-quest (Figure 3 and Table 1). To be specific, we first designed [Ser^23,29^]R2AW to explore the effect of the intra-disulphide bond on the bioactivity of the ranatuerin-2 family. Serine residue possesses chemical properties and a structure similar to cysteine, with the only difference being the terminal groups of hydroxyl and thiol on their respective sidechains. Therefore, a linear mutant was designed where the cysteines on positions 23 and 29 were substituted by serine so that it was unable to form a disulphide bridge. To further confirm whether the cyclic heptapeptide domain is essential for maintaining the function of R2AW, the truncated product R2AW(1-22) was synthesised by removing the Rana box at the C-terminus. Furthermore, considering that the amidation at the C-terminus would facilitate the antimicrobial activity and stability of AMPs, in addition to reducing the potential cytotoxicity [20,21,22], R2AW(1-22)-NH_2_ was designed based on the sequence of truncated derivative R2AW(1-22). As shown in the helical wheel plots (Figure 3D), there are four lysine and two acidic aspartic acids, making the net charge +2 (HeliQuest calculates the net charge at pH = 7.4), and the positively charged residues were relatively dispersed. Considering that the cationicity and hydrophobicity play crucial roles in the function of AMPs [23], [Lys^4,19^, Leu^20^]R2AW(1-22)-NH_2_ was designed from R2AW(1-22)-NH_2_ by substituting aspartic acids in positions 4 and 16 with lysine; meanwhile, a lysine on the hydrophobic side was replaced with leucine. To further explore the effect of hydrophobicity on the function of the peptide, [Trp^6,10^]R2AW(1-22)-NH_2_ was synthesised. The hydrophobic tryptophan residue with a bulky indole sidechain was reported to favour insertion into lipid bilayers [24,25]. Therefore, two alanine residues on positions 6 and 10 were replaced with tryptophan residues. Table 1 summarises the physicochemical properties of R2AW and its five analogues, including peptide sequence, hydrophobicity, hydrophobic moment, and net charge. 

### 2.3. Conformational Analysis of the Designed Analogues of R2AW

**Figure 4:**
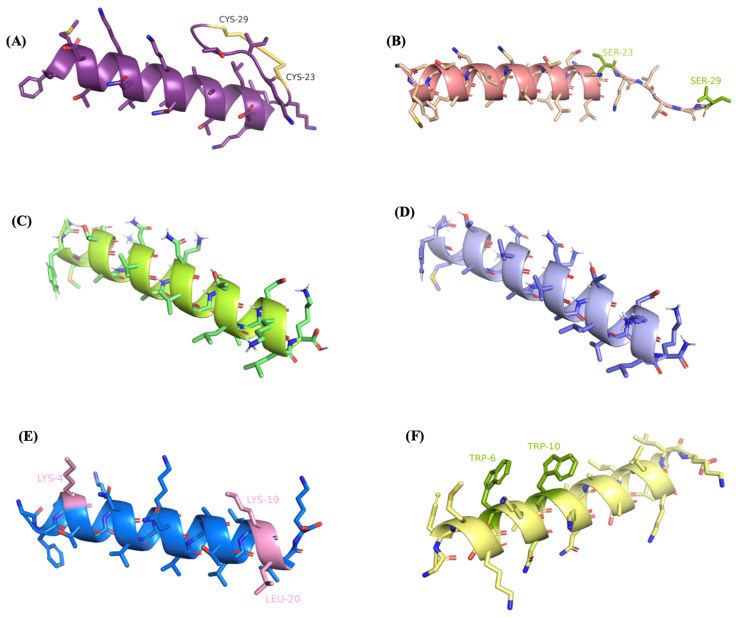
Secondary structure predictions and visualisations of (**A**) R2AW, (**B**) [Ser^23,29^]R2AW, (**C**) R2AW(1-22), (**D**) R2AW(1-22)-NH_2_, (**E**) [Lys^4,19^, Leu^20^]R2AW(1-22)-NH_2_, and (**F**) [Trp^6,10^]R2AW(1-22)-NH_2_, using PEPFOLD-3 and PyMOL. The disulphide bridge was formed by cysteines on positions 23 and 29 shown in yellow colour. The modifications of amino acid residues are labeled and highlighted in colours.

The secondary structures of five analogues were predicted and the potential models are shown in Figure 4. A Ramachandran plot is commonly used to evaluate the quality and reliability of the predicted structure [26]. The structure plausibility was assessed through φ (phi) and ψ (psi) angle distributions of the amino acid residues, classifying them into favoured (red), additionally allowed (yellow), generously allowed (pale yellow), and disallowed regions (white) [27,28]. As displayed in Figure S1, there were few residues located in disallowed regions and more than 90% of the amino acids were plotted in the most favoured region, indicating the high quality of the predicted models. 

**Figure 5:**
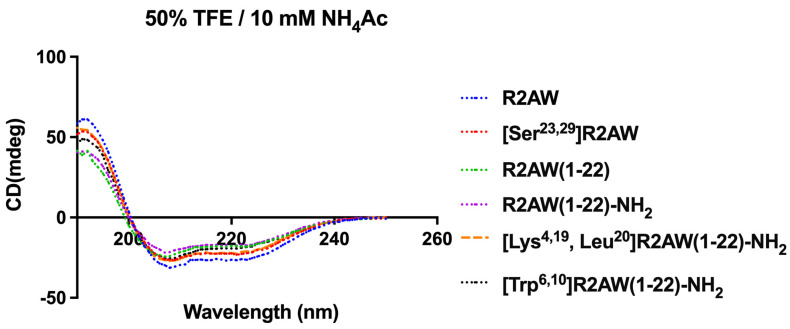
CD spectra of R2AW and its analogues (50 µM) in 50% trifluoroethanol (TFE)/10 mM ammonium acetate (NH_4_Ac) solution.

As shown in Figure 5, the results of circular dichorism (CD) spectroscopy were in accordance with the Pepfold-3 secondary structure prediction. All tested peptides possessed an α-helical structure in 50% trifluoroethanol (TFE).

### 2.4. Minimum Inhibitory Concentration (MIC) and Minimum Bactericidal Concentration (MBC) of Five Analogues of R2AW

The antimicrobial activities of five analogues of R2AW were investigated using six microorganisms, including Gram-positive bacteria *Staphylococcus aureus* (*S. aureus*) (NCTC 10788), MRSA (NCTC 12493), *Enterococcus faecalis* (*E. faecalis*) (NCTC 12697), Gram-negative bacteria *Escherichia coli* (*E. coli*) (ATCC 8379), *Klebsiella pneumoniae* (*K. pneumoniae*) (ATCC 43816), and *Pseudomonas aeruginosa* (*P. aeruginosa*) (ATCC 9027). The minimum inhibitory concentration (MIC) and minimum bactericidal concentration (MBC) values are summarised in Table 2. Generally speaking, R2AW displayed moderate antibacterial activity with a MIC value of 96.3 mg/L (32 µM) against *S. aureus* and *E. coli*. The MIC of the peptide [Ser^23,29^]R2AW (lacking a Rana box) against *S. aureus* and *E. coli* was 190 mg/L (64 µM). In addition, [Ser^23,29^]R2AW could only inhibit the growth of MRSA, *K. pneumoniae,* and *E. faecium* around the concentration of 762 mg/L (256 µM). It is worth noticing that R2AW(1-22) showed no antibacterial activity against these microorganisms after the extraction of the cyclic heptapeptide Rana box without amidation. In contrast, the truncated product R2AW(1-22)-NH_2_ with C-terminal amidation showed similar antibacterial activity to R2AW and [Ser^23,29^]R2AW, indicating that the Rana box was indispensable for antibacterial activity. Surprisingly, the cationicity- and hydrophobicity-enhanced product, [Lys^4,19^, Leu^20^]R2AW(1-22)-NH_2_, exhibited remarkable antibacterial activity. In particular, the activity against Gram-positive bacteria and Gram-negative bacteria was optimised sharply with MIC values ranging from 4.7 mg/L (2 µM) to 18.9 mg/L (8 µM). Interestingly, the antimicrobial activity of the double tryptophan-substituted peptide [Trp^6,10^]R2AW(1-22)-NH_2_ was decreased compared to [Lys^4,19^, Leu^20^]R2AW(1-22)-NH_2_.

### 2.5. Prevention and Eradication of Biofilm by Five Designed Analogues of R2AW

The antibiofilm activity was assessed in the presence of R2AW and its analogues. Table 3 summarises the results of the minimum biofilm inhibitory concentration (MBIC) and minimum biofilm eradication concentration (MBEC). The serine-substitution product [Ser^23,29^]R2AW showed slight antibiofilm activity with an MBIC of 128 μM against *S. aureus* and *E. coli*. As for the truncated peptide R2AW(1-22), it failed to have any activity against the formation of biofilm. However, after C-terminal amidation, R2AW(1-22)-NH_2_ displayed comparable antibiofilm activity to R2AW and [Ser^23,29^]R2AW. Therefore, it was concluded that the Rana box and cysteine bridge were dispensable for the antibiofilm activity of R2AW. It is worth noticing that the cationicity- and hydrophobicity-enhanced peptide, [Lys^4,19^, Leu^20^]R2AW(1-22)-NH_2_, possessed the most potent activity against biofilm formation among all tested peptides, with an MBIC value ranging from 4 μM to 16 μM, whereas [Lys^4,19^, Leu^20^]R2AW(1-22)-NH_2_ could only eradicate the formed biofilm at a concentration around 256 μM. It was found that the tryptophan-replacement peptide, [Trp^6,10^]R2AW(1-22)-NH_2_, had similar antibiofilm activity to [Lys^4,19^, Leu^20^]R2AW(1-22)-NH_2_, which suggested that the continued increase in hydrophobicity failed to benefit the antibacterial activity.

### 2.6. Killing Kinetics against S. aureus, E. coli, MRSA, and P. aeruginosa by [Lys^4,19^, Leu^20^]R2AW(1-22)-NH_2_

**Figure 6:**
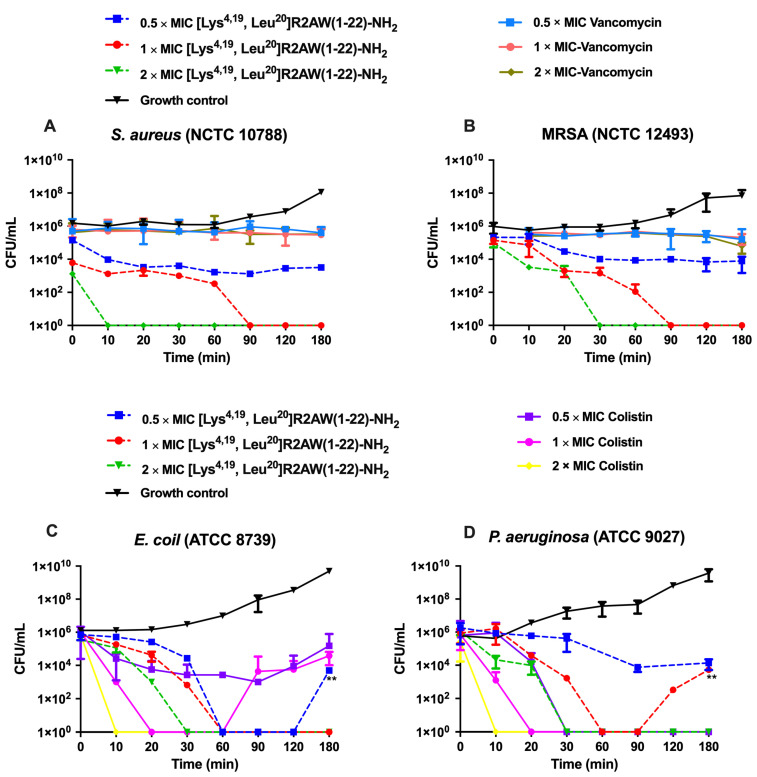
The kinetic time–killing curves of [Lys^4,19^, Leu^20^]R2AW(1-22)-NH_2_ against (**A**) *S. aureus*, (**B**) MRSA, (**C**) *E. coli*, and (**D**) *P. aeruginosa* at concentrations of 0.5 × MIC, 1 × MIC, 2 × MIC. The peptide was added at 0 min and monitored until 180 min. Vancomycin was used as a traditional antibiotic control for Gram-positive bacteria, and colistin was used as a peptide control for Gram-negative bacteria. The CFU/mL of *E. coli* in the presence of 0.5 × MIC of [Lys^4,19^, Leu^20^]R2AW(1-22)-NH_2_ at 180 min was compared with that at 90 min by *t*-test. The CFU/mL of *P. aeruginosa* in the presence of 1 × MIC of [Lys^4,19^, Leu^20^]R2AW(1-22)-NH_2_ at 180 min was compared with that at 90 min using a *t*-test. The level of significance was ** *p*< 0.01.

Since [Lys^4,19^, Leu^20^]R2AW(1-22)-NH_2_ showed an ideal broad-spectrum antibacterial activity, the time–killing assay was conducted to assess the time of killing by different concentrations of [Lys^4,19^, Leu^20^]R2AW(1-22)-NH_2_ acting on *S. aureus* (NCTC 10788), *E. coli* (ATCC 8739), MRSA (NCTC 12493), and *P. aeruginosa* (ATCC 9027). As shown in Figure 6, [Lys^4,19^, Leu^20^]R2AW(1-22)-NH_2_ and colistin (a peptide drug) had a rapid bacterial killing effect and showed a concentration dependence. However, the traditional antibiotic, Vancomycin, could only inhibit the growth of bacteria during a 3 h incubation. To be specific, the population of *S. aureus* decreased stably with the treatment of [Lys^4,19^, Leu^20^]R2AW(1-22)-NH_2_ at 0.5 × MIC. Regarding 2 × MIC and 1 × MIC, no surviving bacteria could be counted in the presence of [Lys^4,19^, Leu^20^]R2AW(1-22)-NH_2_ after 10 min and 90 min incubations. As for the resistant strain MRSA (Figure 6B), the killing speed was quite similar, but it took an extra 20 min for [Lys^4,19^, Leu^20^]R2AW(1-22)-NH_2_ to kill all bacteria at the concentration of 2 × MIC. As for Gram-negative bacteria, the number of *E. coli* fell off remarkably during 0–60 min, while 2 × MIC and 1 × MIC of [Lys^4,19^, Leu^20^]R2AW(1-22)-NH_2_ removed all bacteria at 30 min and 60 min, respectively. Interestingly, a significant difference was obtained by comparing the CFU/mL of *E. coli* in the presence of 0.5 × MIC of [Lys^4,19^, Leu^20^]R2AW(1-22)-NH_2_ at 180 min with that at 90 min (shown in Figure 6C), indicating that the population of *E. coli* had recovered after a 3 h incubation. As shown in Figure 6D, *P. aeruginosa* was killed within 30 min at 2 × MIC after exposure to [Lys^4,19^, Leu^20^]R2AW(1-22)-NH_2_. As for 1 × MIC, the bacteria population reduced stably during the first 60 min, then recovered in the next hour.

### 2.7. Membrane Permeability of S. aureus, E. coli, MRSA and P. aeruginosa by [Lys^4,19^, Leu^20^]R2AW(1-22)-NH_2_

**Figure 7:**
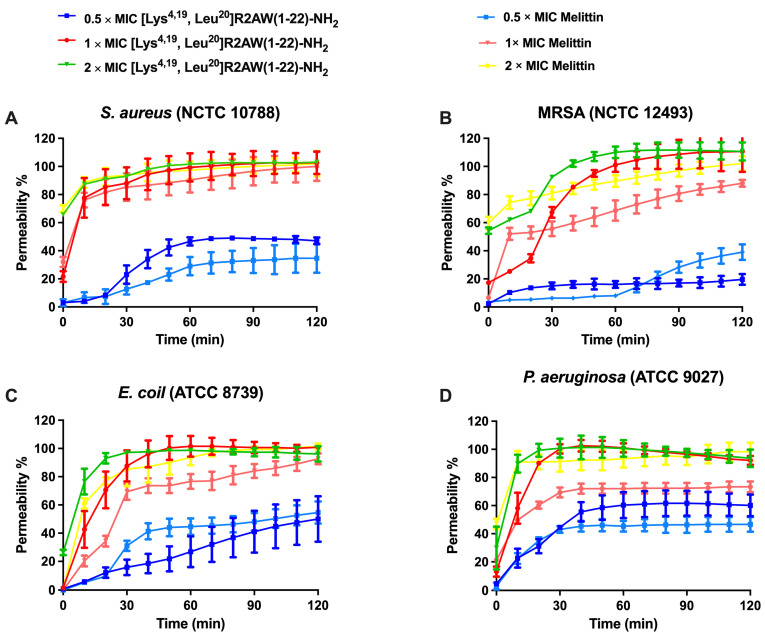
The kinetic membrane permeability curves of [Lys^4,19^, Leu^20^]R2AW(1-22)-NH_2_ and melittin (pore-formation peptide model) against (**A**) *S. aureus*, (**B**) MRSA, (**C**) *E. coli*, and (**D**) *P. aeruginosa* at concentrations of 0.5 × MIC, 1 × MIC, 2 × MIC, and 4 × MIC. The fluorescence was measured every 10 min. The error bar represents the standard error of mean (SEM) of six replicates. The percentage permeability was obtained by comparing the fluorescent intensity of a bacterial suspension treated with 70% isopropanol.

The permeabilizing effect of [Lys^4,19^, Leu^20^]R2AW(1-22)-NH_2_ on *S. aureus* (NCTC 10788), *E. coli* (ATCC 8739), MRSA (NCTC 12493), and *P. aeruginosa* (ATCC 9027) was analysed using the SYTOX Green nucleic acid stain. As shown in Figure 7, [Lys^4,19^, Leu^20^]R2AW(1-22)-NH_2_ and melittin (a pore-formation peptide model) induced membrane permeabilization of these bacteria in a concentration-dependent manner. Generally, the integrity of the bacterial membrane was destroyed rapidly and completely after 30 min exposure to 2 × MIC of [Lys^4,19^, Leu^20^]R2AW(1-22)-NH_2_. In contrast, at 0.5 × MIC, [Lys^4,19^, Leu^20^]R2AW(1-22)-NH_2_ could partially penetrate the membranes of *S. aureus* and MRSA. It is worth noting that the permeabilization rate on *S. aureus* increased from 20% to about 100% after 30 min treatment with [Lys^4,19^, Leu^20^]R2AW(1-22)-NH_2_ at 2 μM, while on MRSA it took an extra 30 min to achieve complete membrane penetration. For *E. coli* and *P. aeruginosa*, the percentage of membrane destruction increased rapidly in the presence of 1 × MIC and 2 × MIC from 0 min to 30 min. In comparison, 0.5 × MIC of [Lys^4,19^, Leu^20^]R2AW(1-22)-NH_2_ exhibited weak membrane activity during that period, followed by a gradual climb over time, which showed similarity with melittin. 

### 2.8. Treatment of MRSA-Infected Waxworms with [Lys^4,19^, Leu^20^]R2AW(1-22)-NH_2_

**Figure 8:**
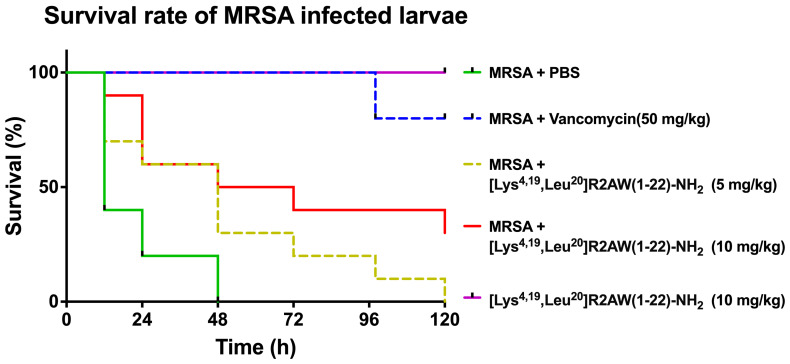
The survival percentage of MRSA-infected waxworms. The larvae were treated with different doses of [Lys^4,19^, Leu^20^]R2AW(1-22)-NH_2_ (5, 10 mg/kg). Phosphate-buffered saline (PBS) and 50 mg/kg of vancomycin were used as negative control and positive control, respectively. Larvae with no MRSA treatment were injected with 10 mg/kg of [Lys^4,19^, Leu^20^]R2AW(1-22)-NH_2_ to evaluate its potential toxicity.

In the in vitro study, [Lys^4,19^, Leu^20^]R2AW(1-22)-NH_2_ had the best antibacterial activity among five designed peptides of R2AW. In this case, we applied an MRSA (NCTC 12493)-infected *Galleria mellonella* larvae model to further evaluate its in vivo antibacterial efficacy. As shown in Figure 8, the mortality of larvae that had been infected with MRSA declined substantially after the treatment with [Lys^4,19^, Leu^20^]R2AW(1-22)-NH_2_. Notably, no death of the larvae was observed in the presence of [Lys^4,19^, Leu^20^]R2AW(1-22)-NH_2_. It was found that about 40% of larvae infected by MRSA survived at the dose of 10 mg/kg over five days. After a lower-concentration (5 mg/kg) treatment, the survival rate of infected larvae was around 10%.

### 2.9. Antiproliferative Activity of Designed Analogues of R2AW against Cancer Cells

**Figure 9:**
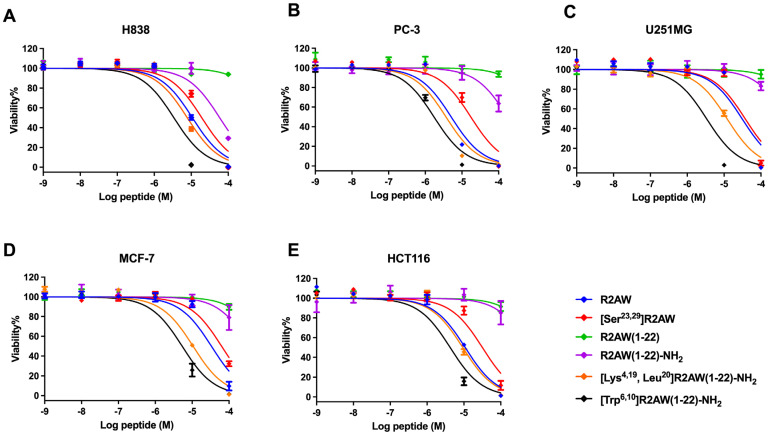
The effect of R2AW and its analogues on the proliferation of (**A**) human prostate carcinoma (PC-3) cell line, (**B**) human non-small-cell lung cancer (H838) cell line, (**C**) human glioblastoma astrocytoma (U251MG) cell line, (**D**) human breast cancer (MCF-7) cell line, and (**E**) human colorectal carcinoma (HCT 116) cell line. Treatment with 0.1% Triton x-100 was used as a positive control. The curves were fitted using normalised dose–response analysis. Error bars indicated the SEM of nine replications.

The antiproliferative activity of designed analogues of R2AW was evaluated on five human cancer cell lines, including non-small-cell lung cancer (H838), human prostate carcinoma (PC-3), human neuronal glioblastoma (U251MG), human breast cancer cell (MCF-7), and colorectal carcinoma (HCT116), at concentrations ranging from 10^−9^ M to 10^−4^ M (Figure 9). Overall, the parent peptide R2AW inhibited the proliferation of cancer cell lines with half-maximal inhibitory concentration (IC_50_) values ranging from 5.093 µM to 33.20 µM (Table 4), while [Ser^23,29^]R2AW was the first designed peptide with the absence of disulphide bridges and this resulted in a slightly decreased activity against the growth of cancer cells. Also, it was found that the antiproliferative activity of truncated peptide R2AW(1-22) was almost lost. As for R2AW(1-22)-NH_2_, which was designed by substituting the Rana box with an amidated lysine, it could inhibit the growth of cancer cells only at a concentration of 100 µM. Notably, [Lys^4,19^, Leu^20^]R2AW(1-22)-NH_2_ presented a broad-spectrum anticancer activity with IC_50_ values between 3.671 µM and 12.04 µM, after introducing lysine and leucine residues. The antiproliferative activity of [Lys^4,19^, Leu^20^]R2AW(1-22)-NH_2_ was most pronounced on PC-3 prostate cancer cells. It was also found that the hydrophobicity-enhanced peptide [Trp^6,10^]R2AW(1-22)-NH_2_ showed significant inhibition of the growth of cancer cells at 10 µM.

### 2.10. Cell Viability When Using [Lys^4,19^, Leu^20^]R2AW(1-22)-NH_2_ against Human Prostate Cancer Cells

**Figure 10:**
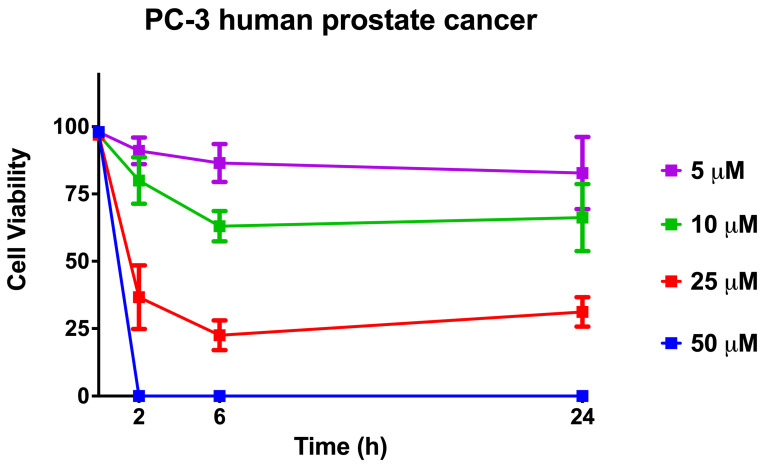
The cell viability when using [Lys^4,19^, Leu^20^]R2AW(1-22)-NH_2_ was evaluated by trypan blue exclusion assay. Human prostate cancer cells PC-3 were treated with the peptide at concentrations of 50 μM, 25 μM, 10 μM, and 5 μM for 2, 6, and 24 h, respectively. The error bars represent the SEM of six replications. The percentage of cell viability was obtained by calculating the ratio of survival cells to total cells.

The cell viability when using [Lys^4,19^, Leu^20^]R2AW(1-22)-NH_2_ on the human prostate cancer cell line, PC-3, was assessed by trypan blue exclusion assay. As shown in Figure 10, the killing effect of the peptide on cancer cells showed a dose-dependent pattern and reached a maximum within 6 h, indicating its strong efficiency. Specifically, 10 μM of [Lys^4,19^, Leu^20^]R2AW(1-22)-NH_2_ caused about 30% cell viability decrease during the period of 0 h to 6 h, while about 75% of prostate cancer cells demonstrated membrane damage after exposure to [Lys^4,19^, Leu^20^]R2AW(1-22)-NH_2_ at a concentration of 25 μM. There were no surviving cells in the presence of 50 μM [Lys^4,19^, Leu^20^]R2AW(1-22)-NH_2_ after 2 h of exposure.

### 2.11. Haemolytic Activities of R2AW and Its Designed Analogues

**Figure 11:**
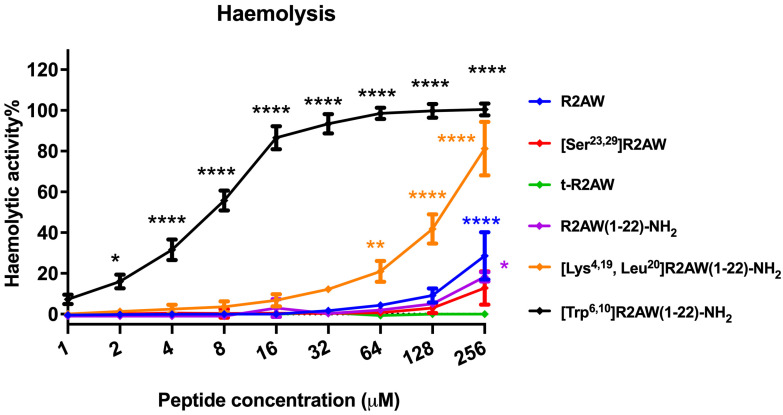
The haemolytic activities of R2AW and its analogues were evaluated using horse erythrocytes at peptide concentrations from 1 μM to 256 μM. Red blood cells treated with 0.1% Triton X-100 or PBS were regarded as positive control and negative control, respectively. The statistical significance was analysed using two-way ANOVA with Dunnett’s multiple comparisons test in GraphPad Prism software by comparison with the negative control (* *p* < 0.05, ** *p* < 0.01, **** *p* < 0.0001). The error bar represents the SEM of nine replications.

The haemolytic activity of five designed variants of R2AW was tested by the employment of a 2% (*v*/*v*) horse erythrocyte suspension. The results are shown in Figure 11. The parent peptide caused about 20% haemolysis up to 256 μM, while [Ser^23,29^]R2AW and the truncated product R2AW(1-22)-NH_2_ showed lower haemolysis at around 10%. As for R2AW(1-22), no haemolysis was observed after the removal of the Rana box without amidation. It is notable that the analogue [Lys^4,19^, Leu^20^]R2AW(1-22)-NH_2_, with enhanced antibacterial and anticancer activities, caused no haemolysis at its maximum MBC value (16 μM), whereas [Trp^6,10^]R2AW(1-22)-NH_2_, designed with the addition of two tryptophans, caused the strong haemolytic activity of approximately 20% at 2 μM.

## 3. Discussion

In this study, a novel peptide, R2AW, was isolated and characterised from the skin secretion of *Amolops wuyiensis.* Homology analysis using the BLAST programme and UniProt database suggested that R2AW belongs to the ranatuerin-2 family. Similar to other peptides from this family (Figure 2), R2AW possessed a cyclic heptapeptide domain at the C-terminus, named a Rana box, whereas the primary structure was poorly conserved. The Rana box structure is found in most AMPs from Ranidae frogs. The function of this cyclic domain in helix-loop peptides remains ambiguous and arguable since it varies in different families. There are several statements about the role of the Rana box. On the one hand, positively charged residues concentrated in this domain provide a net charge. On the other hand, the Rana box could facilitate the helical structure and support protease resistance [11]. In previous studies, researchers also pointed out that the Rana box was dispensable in the nigrocin family (e.g., nigrocin-HL) [18]. However, the function of the cyclic heptapeptide fragment in the ranatuerin-2 family has not yet been thoroughly clarified. R2AW possessed low haemolytic activity and moderate antibacterial activity, similar to other peptides from the ranatuerin-2 family, such as ranatuerin-2PLx [16]. Thus, five analogues were rationally designed to investigate the structural features of R2AW with the hope of improving both its antibacterial and anticancer activities.

The first analogue, [Ser^23,29^]R2AW, was designed to have no intra-disulphide bond by substituting two cysteines at positions 23 and 29 with serine, which is relatively similar to cysteine. Currently, there is no agreement on whether the disulphide bond is indispensable for bioactivity. In this study, it was found that [Ser^23,29^]R2AW possessed a similar antibacterial activity in comparison to the parent peptide, while the anticancer activity was reduced slightly. This result suggested that the absence of disulphide bonds did not affect the antibacterial activity. In addition, it was observed that the substitution of cysteine with serine slightly decreased the helix content. Previous studies also showed that the reduction of intra-disulphide bonds, or replacing cysteine with serine into linear structures, would not affect the antibacterial activity but would reduce haemolysis [17,29], which is consistent with the results of this research. 

Secondly, to confirm whether the cyclic heptapeptide domain is essential for maintaining the function of R2AW, the truncated product R2AW(1-22) was synthesised. Our study showed that the removal of the Rana box domain resulted in the reduction of helical content as well as bioactivities. This cyclic heptapeptide region adopts a helical loop-like fold stably constrained by the disulphide bridge, which is thought to stabilise the a-helical structure of AMPs. The loss of antimicrobial activity observed after removing the Rana box could be attributed to its crucial role in maintaining the helical stability and membrane binding affinity [11,30]. 

Furthermore, we also synthesised an amidated R2AW truncated derivative, R2AW(1-22)-NH_2_, for better comparison. The CD spectra revealed nearly identical helical content in R2AW(1-22) and R2AW(1-22)-NH_2_, suggesting minimal impact of C-terminal amidation on α-helical structure. As stated in the previous research [31,32], helical structure in AMPs is primarily sequence- and composition-dependent, with some AMPs maintaining helix content without C-terminal amidation due to intrinsic helix-forming ability. However, that does not negate the potential influence of amidated and unamidated C-terminuses on the functions of AMPs. Our results indicated that R2AW(1-22)-NH_2_ retained antibacterial activity, which showed similarity to [Ser^23,29^]R2AW. C-terminal amidation alters the surface charge and hydrophobicity of antimicrobial peptides, modulating their interactions with cell membranes [33]. As reported, the amide group enhances adsorption and penetration abilities by forming hydrogen bonds and electrostatic interactions with membrane phospholipid molecules [20,22]. Moreover, amidated peptides have a higher net charge compared to those with an unmodified C-terminus, potentially accounting for their increased activity [21]. Therefore, amidation can compensate for activity loss due to Rana box removal, possibly by enhancing peptide–bacterial membrane interactions, thus surpassing the negative impact of the absence of this cyclic heptapeptide domain. In other words, the artificial elimination of the Rana box in the ranatuerin-2 family did not affect the antibacterial activity after C-terminal amidation. Notably, the antiproliferative activity of R2AW(1-22)-NH_2_ decreased significantly with the absence of a Rana box. Although the reason is not apparent, it was speculated that this fragment affects the affinity of the peptide to cancer cells, making it indispensable.

It has been reported that the physicochemical properties of AMPs control their performance, like net charge and hydrophobicity, both of which have a significant impact on peptide potency [23]. Therefore, a net-charge- and hydrophobicity-enhanced product, [Lys^4,19^, Leu^20^]R2AW(1-22)-NH_2_, was designed based on the truncated sequence with the hope of optimising the antibacterial and anticancer activity. It was found that the ability of the modified product, [Lys^4,19^, Leu^20^]R2AW(1-22)-NH_2_, to inhibit the growth of microorganisms was far more potent than the parent peptide, with MICs ranging from 2 μM to 8 μM towards six different bacteria. Therefore, it was further decided to explore the dynamic killing effect on bacteria. In general, the curves of time–killing and membrane permeability revealed that [Lys^4,19^, Leu^20^]R2AW(1-22)-NH_2_ killed bacteria rapidly through membrane permeabilization. It also acted in a concentration-dependent manner. As shown in Figure 4D and Figure 6D, there was a rising tendency of killing in *E. coli* and *P. aeruginosa* in the presence of 0.5 × MIC and 1 × MIC of [Lys^4,19^, Leu^20^]R2AW(1-22)-NH_2_, respectively. This phenomenon can be explained by the different values of MIC and MBC. The concentration of [Lys^4,19^, Leu^20^]R2AW(1-22)-NH_2_ to kill and inhibit the growth of *E. coli* was the same, but that was not the case for *P. aeruginosa*. Moreover, the concentration of surviving bacteria in the sample treated with the peptide was far too low to be observed in the viable cell counts. According to several publications, a higher density of peptide will accumulate on the lipid membrane, acting as a carpet, thus causing membrane lysis. However, a smaller number of smaller-sized toroidal pores were most likely created at lower peptide concentrations [34]. In this case, [Lys^4,19^, Leu^20^]R2AW(1-22)-NH_2_ was suspected of acting in the same way. In other words, AMPs, in the absence of the target-specific receptor on the surface of microorganisms, displayed a lower possibility of developing resistance compared to conventional antibiotics. Regarding the promising antibacterial activity of [Lys^4,19^, Leu^20^]R2AW(1-22)-NH_2_, the in vivo efficacy was accessed in the MRSA-infected *Galleria mellonella* larvae (waxworm) model [35]. [Lys^4,19^, Leu^20^]R2AW(1-22)-NH_2_ could decrease the mortality rate of infected waxworms, but it was less effective than existing antibiotics, which could be attributed to its low stability. Furthermore, it was indicated that [Lys^4,19^, Leu^20^]R2AW(1-22)-NH_2_ has no toxicityin vivo, which provided a guideline for further in vivo research. Apart from the excellent antibacterial activity, the potency of [Lys^4,19^, Leu^20^]R2AW(1-22)-NH_2_ against the proliferation of cancer cells was evaluated. Generally, [Lys^4,19^, Leu^20^]R2AW(1-22)-NH_2_ showed significance against five human cancer cell lines, especially human prostate cancer cells, at a concentration of 10 µM. According to the trypan blue exclusion assay, [Lys^4,19^, Leu^20^]R2AW(1-22)-NH_2_ only induced partial cytotoxicity at 25 µM. It is reasonable to speculate that there is another mechanism whereby the antiproliferation effects on cancer cells can be achieved without cell membrane disruption, which needs to be further evaluated. Also, the cytotoxicity of [Lys^4,19^, Leu^20^]R2AW(1-22)-NH_2_ should be assessed on normal human cells in future work. In summary, the results showed that the function of R2AW was enhanced significantly after increasing the net charge and hydrophobicity by swapping two acidic amino acids, aspartic acid, with lysine and introducing a leucine residue. To some extent, peptides with positive charges can electrostatically bind with the negatively charged membranes of microorganisms [36,37]. Specifically, the anionic cell wall teichoic acids (WTAs) and lipoteichoic acids (LTAs), attached to the peptidoglycans in Gram-positive bacteria and Gram-negative bacteria, have an outer membrane composed of lipopolysaccharide, making them preferable for AMP binding [38]. In the same way, the membrane of a cancer cell typically has a negative charge because of the high number of anionic molecules like phosphatidylserine, O-glycosidic mucins, and sialylated gangliosides, which could facilitate this interaction [39]. Regarding hydrophobicity, generally, the property of hydrophobicity in AMPs determines the possible extent of penetration of the lipid bilayer leading to membrane disintegration [40].

To further explore the effect of hydrophobicity on the function of the peptide, [Trp^6,10^]R2AW(1-22)-NH_2_ was synthesised by substituting two alanine residues with tryptophan residues. The results indicated that the antibacterial activity of [Trp^6,10^]R2AW(1-22)-NH_2_ was slightly decreased compared to [Lys^4,19^, Leu^20^]R2AW(1-22)-NH_2_. Though [Trp^6,10^]R2AW(1-22)-NH_2_ exhibited the most potent negative effects on the growth of cancer cells, its strong haemolysis limited applications in the clinic. Experiments on the membrane simulation model demonstrated that higher hydrophobicity produces both efficiency and cytotoxicity [41]. Nevertheless, a previous study also indicated a threshold hydrophobicity at which better bioactivity could be achieved [42]. The results here showed that the substitution with tryptophan led to excessive hydrophobicity, which produced an elevated potency against pathogens but also caused damage to host cells.

In conclusion, this study indicated that the antibacterial activity of ranatuerin-2 family peptides could be enhanced via the substitution of acidic amino acids with positively charged lysine residues and the introduction of the leucine on the hydrophobic surface after the removal of the Rana box. Meanwhile, the designed peptide [Lys^4,19^, Leu^20^]R2AW(1-22)-NH_2_, with significantly improved activity and a potential in vivo therapeutic effect against MRSA, could be a rational subject for further study toward clinical applications.

## 4. Materials and Methods

### 4.1. Acquisition of Frog Skin Secretion

Specimens of the frogs, *Amolops wuyiensis* (*n* = 3), were obtained from a commercial supplier in the United Kingdom. All the frogs were adults, and they were kept for at least four weeks in a specialised tropical amphibian facility at Queen’s University Belfast, where they were fed multivitamin-loaded crickets every 2 days and maintained on a 12 h/12 h day and night cycle at 25 °C. The frog skin secretions were collected through gentle electrical stimulation (5V, 100 Hz, and 140 ms pulse width). Subsequently, the stimulated viscous secretions were washed into a cold beaker with deionized water, snap frozen in liquid nitrogen, and then lyophilized. Before analysis, the samples were stored at −20 °C. 

### 4.2. ‘Shotgun’ Cloning and Sequencing of Ranatuerin-2-AW Precursor-Encoding cDNA

Five mg of lyophilised *Amolops wuyiensis* skin secretion was dissolved in 1 mL of Lysis/Binding buffer. A Dynabeads^®^ mRNA DIRECT™ Kit (Dynal Biotech, Merseyside, UK) was used for mRNA isolation. The cDNA library construction was performed using Clontech SMARTer^®^ RACE 5′/3′ Kit (Takara Bio, USA, Inc., San Jose, CA, USA). A nested universal primer (NUP) and a degenerate sense primer (S1: 5′-GAWYYAYYHRAGCCYAAADATG-3′; W = A + T, Y = C + T, H = A + C + T, R = A + G, D = A + G + T) were used. The cDNA ends were rapidly amplified by a PCR thermal cycling system with repeated denaturation, annealing, and extension. The products were analysed by gel electrophoresis and then purified using a Hi-Bind DNA mini-column (Omega Bio-Tek, Norcross, GA, USA). The process of DNA ligation was performed using a pGEM-T Easy Vector System (Promega Corporation, Madison, WI, USA). After that, the recombinant plasmid DNA was cloned in JM109 high-efficiency competent cells (Promega, Madison, WI, USA). A Big Dye^®^ Terminator v3.1 Cycle Sequencing Kit (Applied Biosystems, Foster City, CA, USA) was used for sequencing reaction and the products were analysed using an ABI3730 automated sequencer (Applied Biosystems, Foster City, CA, USA).

### 4.3. Prediction of Secondary Structure

The secondary structures of the series of analogues of R2AW were predicted using Pepfold-3 (https://mobyle.rpbs.univ-paris-diderot.fr/cgi-bin/portal.py#forms::PEP-FOLD (accessed on 9 March 2023)). Meanwhile, the potential models were visualised by PyMOL software and their quality was validated using Ramachandran plots. Also, the structural parameters were analysed using Heli-quest (https://heliquest.ipmc.cnrs.fr/cgi-bin/ComputParams.py (accessed on 9 March 2023)).

### 4.4. Synthesis of R2AW and Its Five Designed Analogues

The peptides were chemically synthesised by a solid-phase peptide synthesiser (Tribute 2-channel peptide synthesiser, Protein Technologies, Tucson, AZ, USA). The whole synthesis process was described in detail in a previous paper [43]. The amino acids were weighed accurately and mixed with 2-(1H-benzotriazole-1-yl)-1,1,3,3-tetramethyluronium hexafluorophosphate (HBTU). The Rink amide MBHA resin or Fmoc-Cys(Trt)-Wang resin (100–200 mesh) (Millipore Sigma, Burlington, MA, USA) was weighed and loaded into a 15 mL vessel before running the synthesiser. The synthesis process involved a serial reaction cycle of washing, activation, deblocking, and coupling. The resin was washed with dimethylformamide (DMF) (Sigma-Aldrich, Gillingham, UK, 99%) for 5 min, then the Fmoc protecting groups were deprotected using 20% piperidine (Sigma-Aldrich, Gillingham, UK, 99%) in DMF. Next, 11% N-Methylmorpholine (NMM) (Sigma-Aldrich, Gillingham, UK, 99%) in DMF was used to catalyse the coupling reaction. The peptide was synthesised from the C-terminus to the N-terminus. After that, degassed dichloromethane (DCM) was employed for washing the peptide/resin complex after the synthesis reaction. The protected peptide was cleaved from the resin and deprotected by 2 h stirring reaction with the cleavage cocktail (25 mL/1g), including 94% trifluoroacetic acid (TFA), 2% water, 2% triisopropylsilane (TIS), and 2% 1, 2-Ethanedithiol (EDT). The peptides were washed by cold diethyl ether, and then dissolved in buffer A (H_2_O/TFA (99.95/0.05, *v*/*v*). The crude peptides were stored at −20 °C after lyophilisation. 

### 4.5. Purification and Characterisation of R2AW and Its Designed Analogues

The crude peptides were purified using a reverse phase HPLC system (Waters^®^, Milford, MA, USA) that consisted of Waters 1500 Series pumps, a Waters 2489 UV/Visible Detector, and a Phenomenex Aeris 5 µm Peptide Xb-C18 Column 250 × 21.2 mm. The molecular masses of the purified products were obtained and confirmed by matrix-assisted laser desorption/ionisation time-of-flight (MALDI-TOF) mass spectrometry (Perspective Biosystems, Framingham, MA, USA) (Supplemented in Figures S2 and S3). A total of 10 mg/mL of the α-cyano-4-hydroxycinnamic acid (CHCA) was used as the matrix solution. 

### 4.6. Circular Dichroism Spectra

The secondary structures of R2AW and its five analogues were investigated using a JASCO-815 CD spectrometer (JASCO Inc., Tokyo, Japan). Each peptide was dissolved in 50% TFE (*v*/*v* in 10 mM NH_4_Ac (pH = 7)) to a final concentration of 50 µM and then 200 µL of each peptide solution was loaded into a 1 mm path length curette. Samples were analysed within the range of 190 to 250 nm at room temperature at a scanning speed of 100 nm/min, a bandwidth of 1 nm, and a data pitch of 1 nm.

### 4.7. Determination of MICs and MBCs

The MIC and MBC assays were used to assess the inhibitory activities of all 5 analogues of R2AW on the growth of planktonic microorganisms. The MIC is the lowest concentration that prevents the viable growth of bacteria. The MBC is the lowest concentration that results in bacterial death. Six microorganisms were used to detect the antimicrobial activity of peptides, including Gram-positive bacteria *S. aureus* (NCTC 10788), MRSA (NCTC 12493), *Enterococcus faecalis* (*E. faecalis*) (NCTC 12697), Gram-negative bacteria *E. coli* (ATCC 8379), *Klebsiella pneumoniae* (*K. pneumoniae*) (ATCC 43816), and *Pseudomonas aeruginosa* (*P. aeruginosa*) (ATCC 9027). According to the assay described previously [44], the microorganisms were inoculated at 37 °C overnight and diluted to 5 × 10^5^ CFU/mL after reaching the logarithmic growth period. The peptide stock solution at the concentration of 512 × 10^2^ μM was prepared and diluted to 100 μM by a 2-fold dilution. One μL of each concentration of peptide solution was incubated with 99 μL of bacterial subculture in the 96-well plate for 18 h at 37 °C. The absorbance was determined at a wavelength of 550 nm using a Synergy HT plate reader (Bio Tek, Washington, DC, USA). The peptide concentration that resulted in invisible bacteria in the 96-well plate was determined as the MIC. As for those wells that were clearly visible, 20 μL of the solution was dropped onto the Mueller Hinton agar (MHA) dish and then incubated at 37 °C overnight. The lowest concentration with no colony growth was determined as the MBC.

### 4.8. Antibiofilm Assays

As a previous study [45] described in detail, the ability of R2AW and its analogues to inhibit and eradicate the formation of biofilm was investigated. The MBIC is the lowest concentration which inhibits the formation of biofilm. The MBEC is the minimum concentration that eradicates biofilm. Six bacterial strains were used, as described above. Briefly, the bacteria were cultured and then diluted to 5 × 10^5^ CFU/mL. In MBIC assays, 1 μL of each concentration of R2AW and its analogues (ranging from 512 × 10^2^ μM to 100 μM) was mixed with 99 μL of diluted bacterial suspension in a 96-well plate for 16-20 h at 37 °C. As for MBEC assays, 100 μL of cultured bacteria were seeded in a 96-well plate and incubated on the shaking incubator at 37 °C overnight for biofilm formation. Subsequently, each well was rinsed twice with 100 μL sterile PBS to remove planktonic cells, and then replaced with 100 μL of each concentration (ranging from 512 μM to 1 μM) of peptides. After 24 h incubation, the biofilm was washed twice with 100 μL of PBS and fixed with methanol. Next, the same volume of 0.1% crystal violet solution (Sigma-Aldrich, Gillingham, UK) was added to each well to stain the biofilm. After that, 100 μL of 30% of acetic acid (Sigma-Aldrich, Gillingham, UK) was transferred to each well for dissolution. The solutions were transferred into a new 96-well plate and the absorbances of wells was monitored using a Synergy HT plate reader set to 595 nm.

### 4.9. Time–Killing Assays

The time–killing assay was used to determine the kinetic killing of bacteria by various concentrations of [Lys^4,19^, Leu^20^]R2AW(1-22)-NH_2_ that exhibited the best antibacterial activity among the designed peptides. *S. aureus* (NCTC 10788), *E. coli* (ATCC 8739), MRSA (NCTC 12493), and *P. aeruginosa* (ATCC 9027) were used in this assay. Treatments of 2 × MIC, 1 × MIC, and 0.5 × MIC of [Lys^4,19^, Leu^20^]R2AW(1-22)-NH_2_ were prepared and mixed with 198 μL of diluted bacteria (5 × 10^5^ CFU/mL). At each time point (0, 10, 20, 30, 60, 90, 120, and 180 min), 10 μL of peptide and bacteria mixture was dropped onto the MHA plate. After overnight incubation, the number of bacterial colonies was calculated. The CFU/mL could be calculated using the formula CFU/mL = (Number of colonies × dilution factor)/volume of culture.

### 4.10. Bacterial Cell Membrane Permeability Assays

The effect of [Lys^4,19^, Leu^20^]R2AW(1-22)-NH_2_ on membrane integrity of *S. aureus* (NCTC 10788), *E. coli* (ATCC 8739), MRSA (NCTC 12493), and *P. aeruginosa* (ATCC 9027) was studied by applying SYTOX Green nucleic acid stain (Thermo Fisher Scientific, Waltham, MA, USA). These four bacteria were inoculated into a TSB medium and grown to the logarithmic phase. The supernatant was decanted after centrifugation (1000× *g*, 10 min, 4 °C). The bacteria were washed with 5% TSB in 0.85% NaCl solution and resuspended until a 1 × 10^8^ CFU/mL density was reached and assessed by measuring the OD value (0.7) at wavelength 590 nm. Forty µL of [Lys^4,19^, Leu^20^]R2AW(1-22)-NH_2_ at concentrations of 4 × MIC, 2 × MIC, and 1 × MIC was added to a black 96-well plate. Meanwhile, 10 μL of diluted SYTOX green-fluorescent nucleic acid stain (50 µM, Life Technologies, Renfrew, UK) was transferred into each well. Next, 50 μL of the bacterial suspension (1 × 10^8^ CFU/mL) was transferred into each well. The plate was analysed by the Synergy HT plate reader using a 2 h kinetic programme with excitation at 485 nm and emission at 580 nm.

### 4.11. Evaluation of Efficacy of [Lys^4,19^, Leu^20^]R2AW(1-22)-NH_2_ against MRSA in Insect Larvae

According to a previous publication, the in vivo antibacterial activity of [Lys^4,19^, Leu^20^]R2AW(1-22)-NH_2_ was assessed using the larva of *Galleria mellonella* with minor revision [46]. The waxworms (Livefood UK Ltd., Rooks Bridge, UK) were weighed, then larvae (250 ± 50 mg) were picked and used at 10 per plate. The waxworms were injected with 10 μL of MRSA (NCTC12493) suspension (1 × 10^7^ CFU/mL) that was prepared in sterilised PBS. After 1 h of infection, each waxworm was administered 10 μL of either 5 mg/kg or 10 mg/kg peptide solution. The same injection volume of PBS and 50 mg/kg vancomycin were regarded as negative and positive controls, respectively. Each group contained 10 larvae and the numbers of survivors were recorded every 12 h for five days.

### 4.12. Anticancer MTT Assays

Five human cancer cell lines of non-small-cell lung cancer H838, human prostate carcinoma PC-3, human neuronal glioblastoma U251MG, human breast cancer cell MCF-7, and colorectal carcinoma HCT116 were used to detect the antiproliferative abilities of R2AW and its five analogues. According to the procedures in a previous article [47], cells (8000 cells/well) were seeded into the 96-well tissue culture plates and incubated at 37 °C with 5% CO_2_ overnight. The solutions of R2AW and its analogues at concentrations of 10^−4^ M to 10^−9^ M were prepared in the serum-free medium. Next, cells in each well were treated with 100 μL of each peptide solution for 24 h. Subsequently, 10 μL of 5 mg/mL MTT (3-(4, 5-Dimethylthiazol-2-yl-)-2, 5-Diphehyltetrazolium Bromide) was added to each well followed by 37 °C incubation for 2 h. After that, the liquid in each well was removed and 100 μL of DMSO was added to dissolve the purple formazan. The plate was placed on a shaking incubator for 10 min, and the absorbance was detected at 550 nm using a Synergy HT plate reader (Biotech, Minneapolis, MN, USA).

### 4.13. Trypan Blue Exclusion Assays

In the MTT assay, [Lys^4,19^, Leu^20^]R2AW(1-22)-NH_2_ showed the best antiproliferative activity against human prostate cancer cells, PC-3. In this context, the cytotoxicity of [Lys^4,19^, Leu^20^]R2AW(1-22)-NH_2_ on PC-3 cells was determined by trypan blue exclusion assay [43]. Cells (2 × 10^5^ cells/well) were seeded into a 12-well plate and incubated at 37 °C with 5% CO_2_ overnight. After 4 h of starvation, 500 μL of [Lys^4,19^, Leu^20^]R2AW(1-22)-NH_2_ at the concentrations of 50 μM, 25 μM, 10 μM, and 5 μM was added to each well and incubated at 37 °C for 2 h, 6 h, and 24 h. At each time point, the cell suspension of each well was made and transferred to the 1.5 mL tube for centrifugation (380× *g*, 5 min, 18 °C). After removing the supernatant, 400 μL of PBS was added to resuspend the cell pellet. Next, 10 µL of cell suspension was mixed with the same volume of trypan blue (0.4%, Gibco, Brooklyn, NY, USA). Then, 10 μL of the mixture was loaded into a haemocytometer for cell counting. The percentages of viable cells and dead cells were calculated by counting the unstained and stained cells, separately. 

### 4.14. Haemolysis Assays

The haemolytic activity of R2AW and its five designed analogues on mammalian erythrocytes was evaluated using 2% defibrinated horse blood (TCS Biosciences Ltd., Buckingham, UK) as per a previous report [48]. Two mL of fresh horse blood was transferred into a 50 mL tube and then washed with sterilised PBS. The supernatant was removed after centrifuging (930× *g*, 5 min). The centrifugation procedure was repeated until the supernatant was clear. After washing, the red blood cell pellet was diluted with PBS to achieve a 4% (*v*/*v*) erythrocyte suspension. The concentrations of R2AW and its designed derivatives were prepared from 512 μM to 2 μM by two-fold dilution in sterilised PBS. One hundred µL of red blood cell suspension was added into a 1.5 mL tube treated with the same volume of each concentration of peptide and incubated at 37 °C for 2 h. The red blood cell suspensions treated with 0.2% Triton X-100 and with PBS were regarded as positive control and negative control. After centrifugation at 930× *g* for 10 min, 100 μL of suspension was transferred to a 96-well plate, and the absorbance was obtained by use of a Synergy HT plate reader. The degree of haemolysis could be calculated using the following formula: Haemolysis = (absorbance (experimental groups) − absorbance (negative control))/(absorbance (positive control) − absorbance (negative control)) × 100%.

## Figures and Tables

**Table 1:** Structural parameters of R2AW and its analogues. Heli-quest calculates the net charge at pH = 7.4.

Name	Sequence	Hydrophobicity	Hydrophobicity Moment	Net Charge
R2AW	GFMDTAKNVAKNVAATLLDKLKCKITGGC	0.337	0.225	+3
[Ser^23,29^]R2AW	GFMDTAKNVAKNVAATLLDKLKSKITGGS	0.228	0.279	+3
R2AW(1-22)	GFMDTAKNVAKNVAATLLDKLK	0.255	0.412	+2
R2AW(1-22)-NH_2_	GFMDTAKNVAKNVAATLLDKLK-NH_2_	0.255	0.412	+3
[Lys^4,19^, Leu^20^]R2AW(1-22)-NH_2_	GFMKTAKNVAKNVAATLLKLLK-NH_2_	0.358	0.516	+6
[Trp^6,10^]R2AW(1-22)-NH_2_	GFMKTWKNVWKNVAATLLKLLK-NH_2_	0.534	0.674	+6

**Table 2:** MICs and MBCs of R2AW and its analogues against different microorganisms.

Peptides	MIC_s_ (mg/L and µM)					
	*S. aureus* (NCTC 10788)	*E. coli * (ATCC 8739)	MRSA (NCTC 12493)	*K. pneumoniae* (ATCC 43816)	*E. faecium* (NTCC 12697)	*P. aeruginosa* (ATCC 9027)
R2AW	96.3 (32)	96.3 (32)	770 (256)	385 (128)	770 (256)	385 (128)
[Ser^23,29^]R2AW	190 (64)	190 (64)	763 (256)	763 (256)	763 (256)	1526 (512)
R2AW(1-22)	>1203 (>512)	>1203 (>512)	>1203 (>512)	>1203 (>512)	>1203 (>512)	>1203 (>512)
R2AW(1-22)-NH_2_	150 (64)	300 (128)	601 (256)	601 (256)	601 (256)	1202 (512)
[Lys^4,19^, Leu^20^]R2AW(1-22)-NH_2_	4.7 (2)	4.7 (2)	4.7 (2)	9.4 (4)	9.4 (4)	18.9 (8)
[Trp^6,10^]R2AW(1-22)-NH_2_	10.4 (4)	20.7 (8)	10.4 (4)	41.4 (16)	20.7 (8)	82.8 (32)
Vancomycin	0.7 (0.5)	NA *	0.7 (0.5)	NA *	0.7 (0.5)	NA *
Colistin	NA *	0.1 (0.125)	NA *	18.5 (16)	NA *	1.2 (1)
Melittin	5.7 (2)	11.4 (4)	5.7 (2)	91.1 (32)	5.7 (2)	45.5 (16)
	**MBC_s_ (mg/L and µM)**					
R2AW	193 (64)	193 (64)	1541 (512)	771 (256)	771 (256)	1541 (512)
[Ser^23,29^]R2AW	191 (64)	381 (128)	1526 (512)	1526 (512)	1526 (512)	>1526 (>512)
R2AW(1-22)	>1202 (>512)	>1202 (>512)	>1202 (>512)	>1202 (>512)	>1202 (>512)	>1202 (>512)
R2AW(1-22)-NH_2_	150 (64)	301 (128)	601 (256)	601 (256)	601 (256)	>1202 (>512)
[Lys^4,19^, Leu^20^]R2AW(1-22)-NH_2_	4.7 (2)	9.4 (4)	4.7 (2)	18.9 (8)	18.9 (8)	37.7 (16)
[Trp^6,10^]R2AW(1-22)-NH_2_	20.7 (8)	41.4 (16)	20.7 (8)	82.8 (32)	41.4 (16)	166 (64)
Vancomycin	0.7 (0.5)	NA *	0.7 (0.5)	NA *	>92.7 (>64)	NA *
Colistin	NA *	0.3 (0.25)	NA *	18.5 (16)	NA *	2.3 (2)
Melittin	5.7 (2)	11.4 (4)	5.7 (2)	91.1 (32)	5.7 (2)	91.1 (32)

* NA: Not applicable.

**Table 3:** MBICs and MBECs of R2AW and its analogues against different microorganisms.

Peptides	MBIC/MBEC (µM)					
	*S. aureus* (NCTC 10788)	*E. coli * (ATCC 8739)	MRSA (NCTC 12493)	*K. pneumoniae* (ATCC 43816)	*E. faecium* (NTCC 12697)	*P. aeruginosa* (ATCC 9027)
R2AW	128/>512	128/512	256/>512	512/>512	>512	>512
[Ser^23,29^]R2AW	128/>512	128/512	256/>512	>512	>512	>512
R2AW(1-22)	>512	>512	>512	>512	>512	>512
R2AW(1-22)-NH_2_	128/>512	256/512	256/>256	512/>512	>512	>512
[Lys^4,19^,Leu^20^]R2AW(1-22)-NH_2_	4/256	4/256	4/>512	8/256	8/>512	16/512
[Trp^6,10^]R2AW(1-22)-NH_2_	8/512	4/256	8/>512	16/256	8/>512	32/512

**Table 4:** Antiproliferative IC_50_ values of R2AW and its designed analogues against H838, PC-3, U251MG, MCF-7, and HCT116 cancer cells.

Peptides	IC_50_ (µM)				
	H838	PC3	U251MG	MCF-7	HCT116
R2AW	10.78	5.093	30.78	33.20	10.82
[Ser^23,29^]R2AW	19.34	17.44	37.31	62.7	31.64
R2AW(1-22)	1423	1480	1818	894.5	1124
R2AW(1-22)-NH_2_	58.91	194.3	515.8	373.6	609
[Lys^4,19^, Leu^20^]R2AW(1-22)-NH_2_	7.828	3.671	12.40	10.58	9.284
[Trp^6,10^]R2AW(1-22)-NH_2_	3.405	1.730	3.353	5.328	5.375

## Data Availability

Data are contained within the article and supplementary materials.

## Supplementary Materials

The following supporting information can be downloaded at: https://www.mdpi.com/article/10.3390/antibiotics13010005/s1, Figure S1: Ramachandran plot analysis of predictive structure of (**A**) R2AW, (**B**) [Ser^23,29^]R2AW, (**C**) R2AW(1-22), (**D**) R2AW(1-22)-NH_2_, (**E**) [Lys^4,19^, Leu^20^]R2AW(1-22)-NH_2_, and (**F**) [Trp^6,10^]R2AW(1-22)-NH_2_.; Figure S2: Reverse phase HPLC chromatogram of purified peptides (**A**) R2AW, (**B**) [Ser^23,29^]R2AW, (**C**) R2AW(1-22), (**D**) R2AW(1-22)-NH_2_, (**E**) [Lys^4,19^, Leu^20^]R2AW(1-22)-NH_2_, and (**F**) [Trp^6,10^]R2AW(1-22)-NH_2_.; Figure S3: MALDI-TOF mass spectra of (**A**) R2AW, (**B**) [Ser^23,29^]R2AW, (**C**) R2AW(1-22), (**D**) R2AW(1-22)-NH_2_, (**E**) [Lys^4,19^, Leu^20^]R2AW(1-22)-NH_2_, and (**F**) [Trp^6,10^]R2AW(1-22)-NH_2_.
