# Supplementary material for: Progressive Design of a Ranatuerin-2 Peptide from Amolops wuyiensis: Enhancement of Bioactivity and In Vivo Efficacy
Source: Antibiotics (Basel). 2023 Dec 19;13(1):5. doi: 10.3390/antibiotics13010005 (PMC10812557; doi:10.3390/antibiotics13010005)
Supplement: Supplementary file 1 [file antibiotics-13-00005-s001.zip › antibiotics-2706791-supplementary.pdf]

Supplementary Materials

Progressive design of a ranatuerin-2 peptide from *Amolops wuyiensis*: enhancement of bioactivity and in vivo efficacy

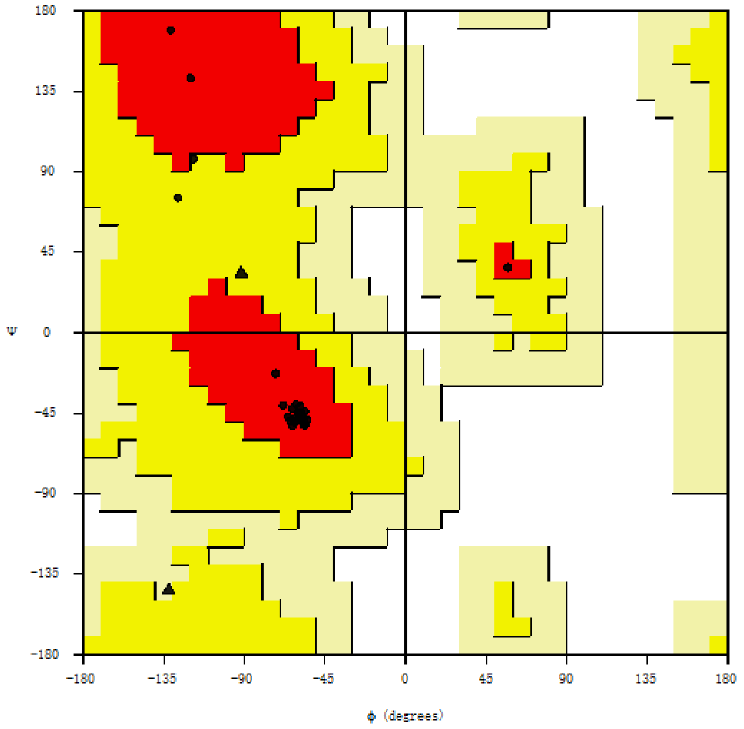

(A)

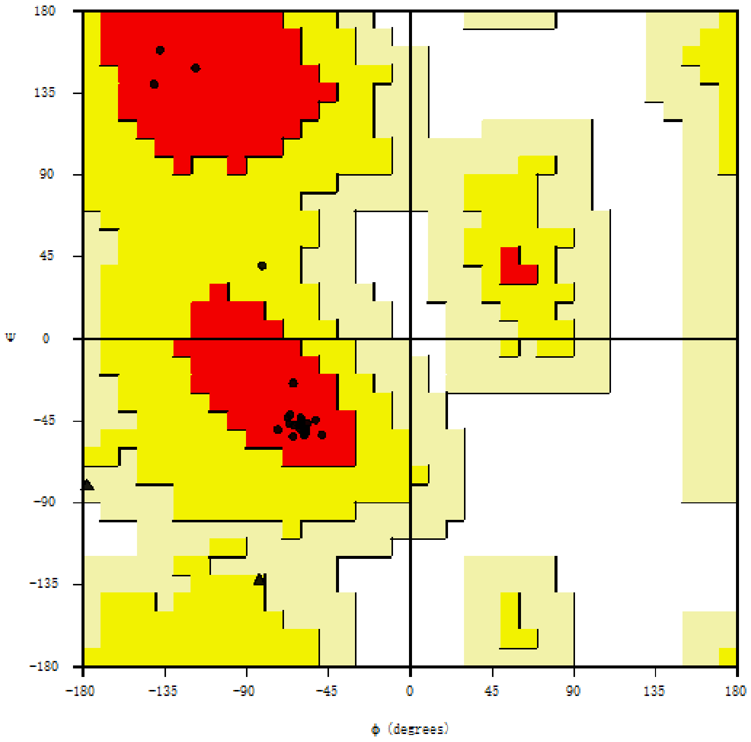

(B)

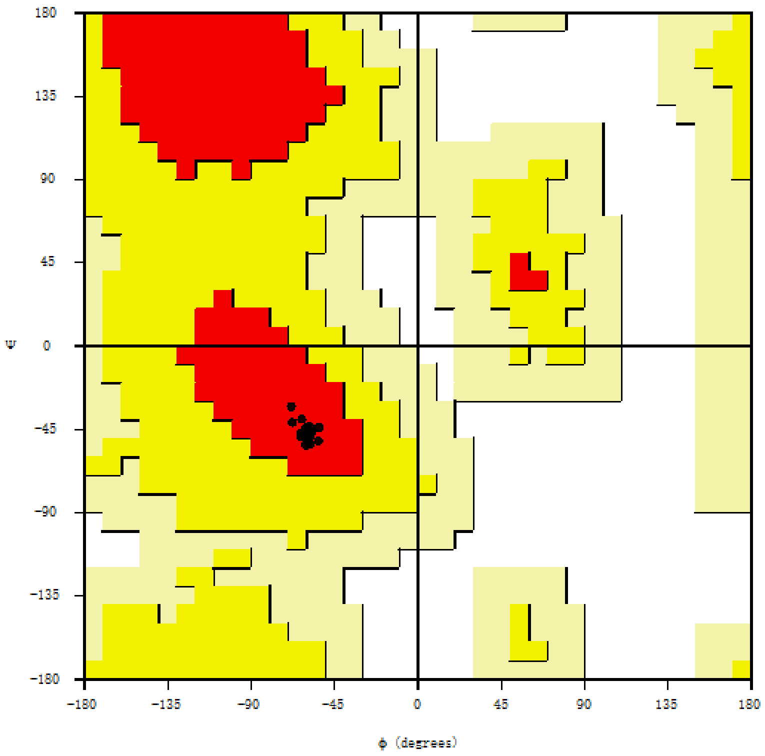

180
135
90
45
0
-45
-90
-135
-180
Ψ
-180
-135
-90
-45
0
45
90
135
180
ϕ (degrees)

(C)

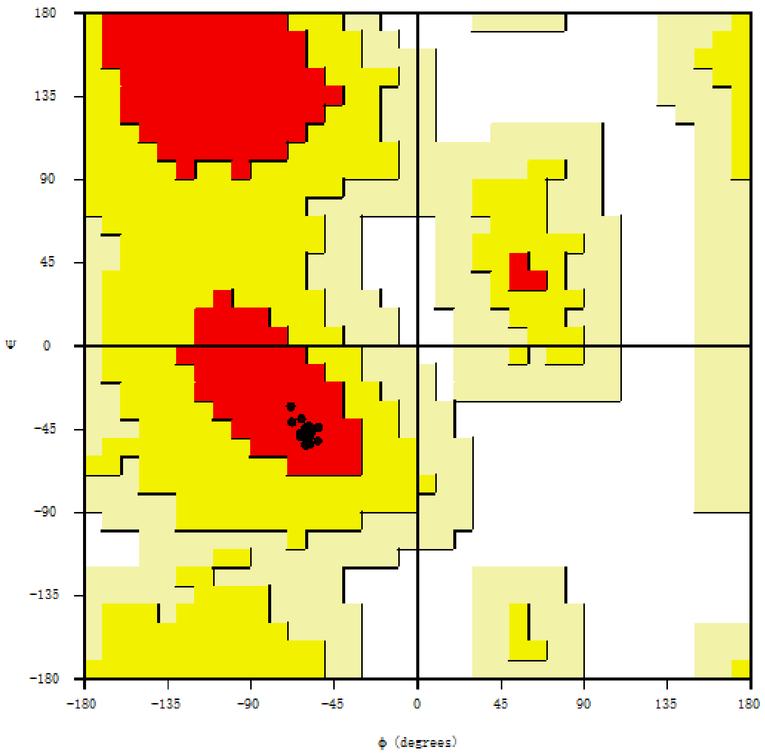

180
135
90
45
0
-45
-90
-135
-180
Ψ
-180
-135
-90
-45
0
45
90
135
180
ϕ (degrees)

(D)

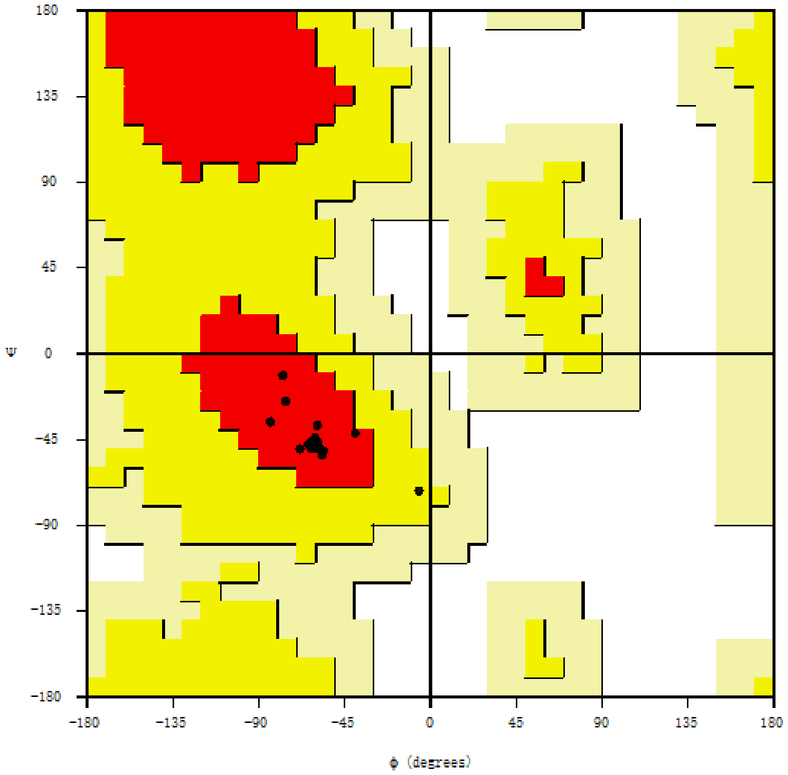

(E)

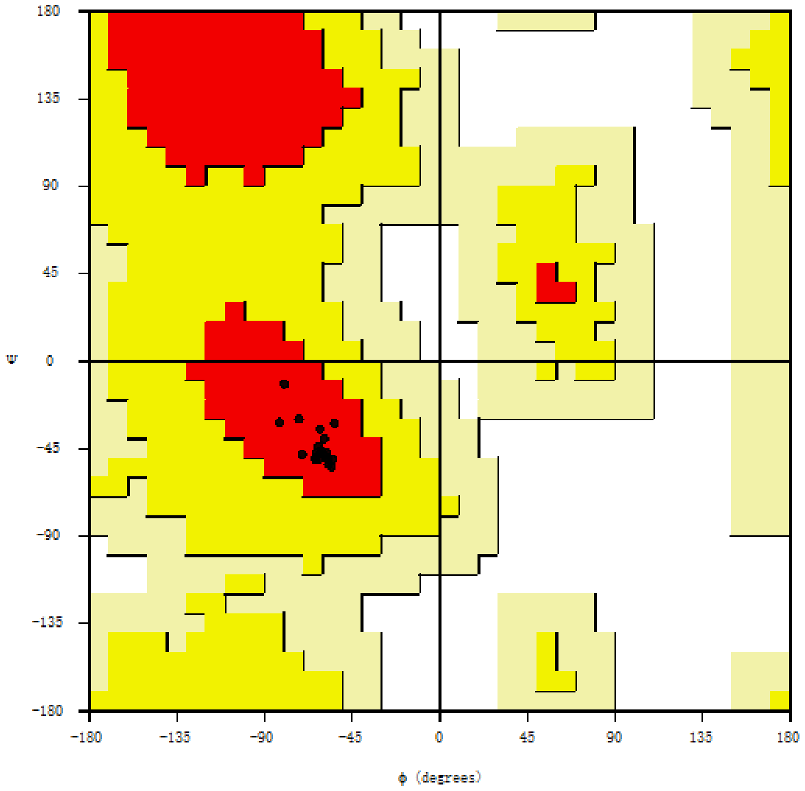

(F)

Figure S1. Ramachandran plot analysis of predictive structure of (**A**) R2AW, (**B**) [Ser^{23,29}]R2AW, (**C**) R2AW(1-22), (**D**) R2AW(1-22)-NH$_2$, (**E**) [Lys^{4,19}, Leu^{20}]R2AW(1-22)-NH$_2$, and (**F**) [Trp^{6,10}]R2AW(1-22)-NH$_2$. It depicts φ (phi) and ψ (psi) angles of peptide units, representing backbone dihedral angles Cα-N-Cα-C and N-Cα-C-N, respectively. Both angles span from -180° to +180°. The Ramachandran diagram divides the φ and ψ angles of amino acid residues into different regions, including most favoured (red), additionally allowed (yellow), generously allowed (pale yellow), and disallowed regions (white). The results indicated good quality of each peptide model with the most favoured region above 90%.

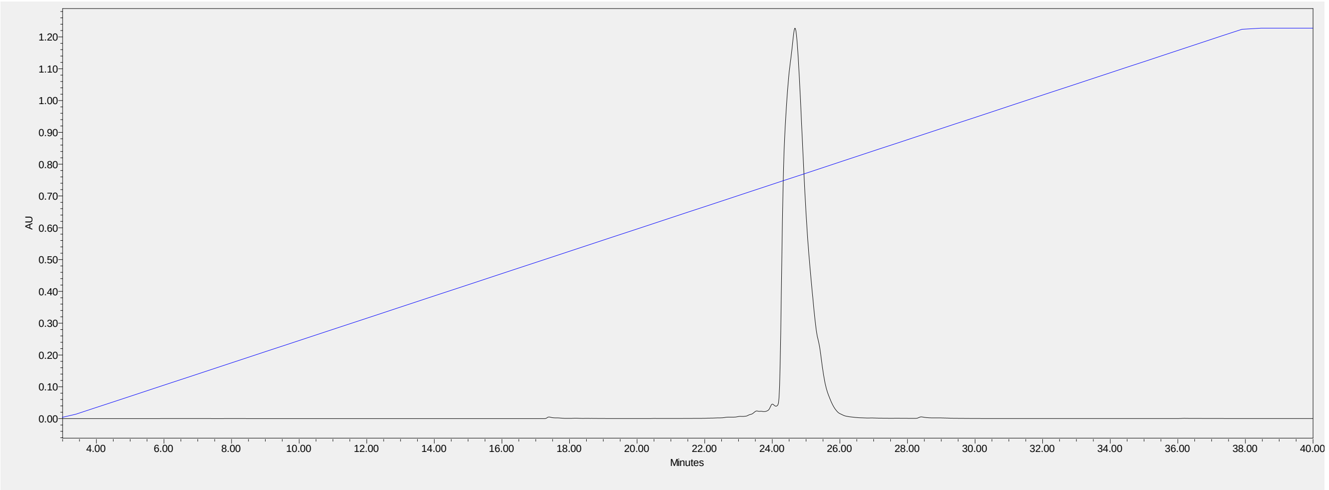

(A)

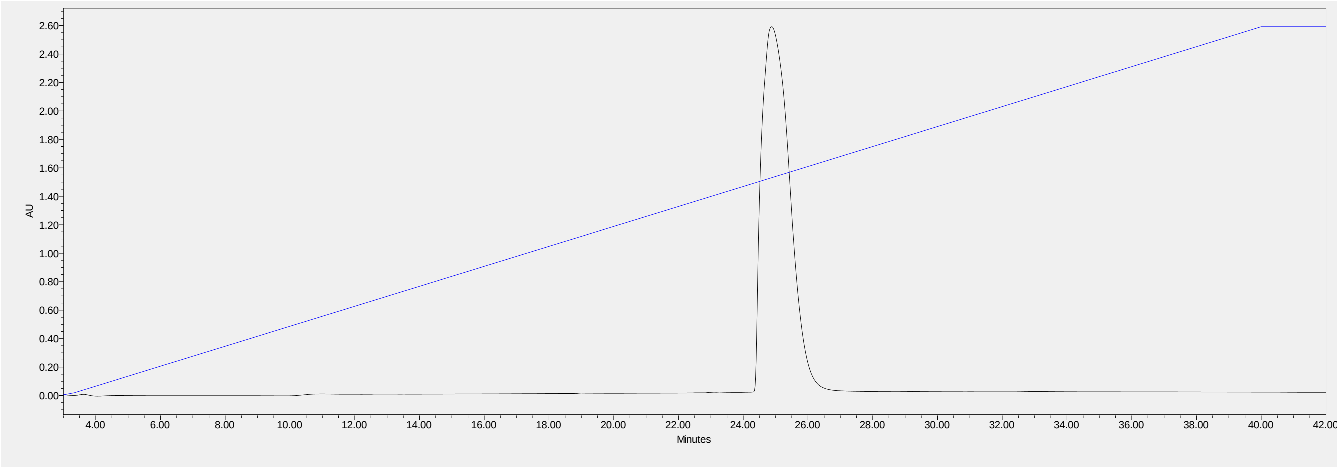

(B)

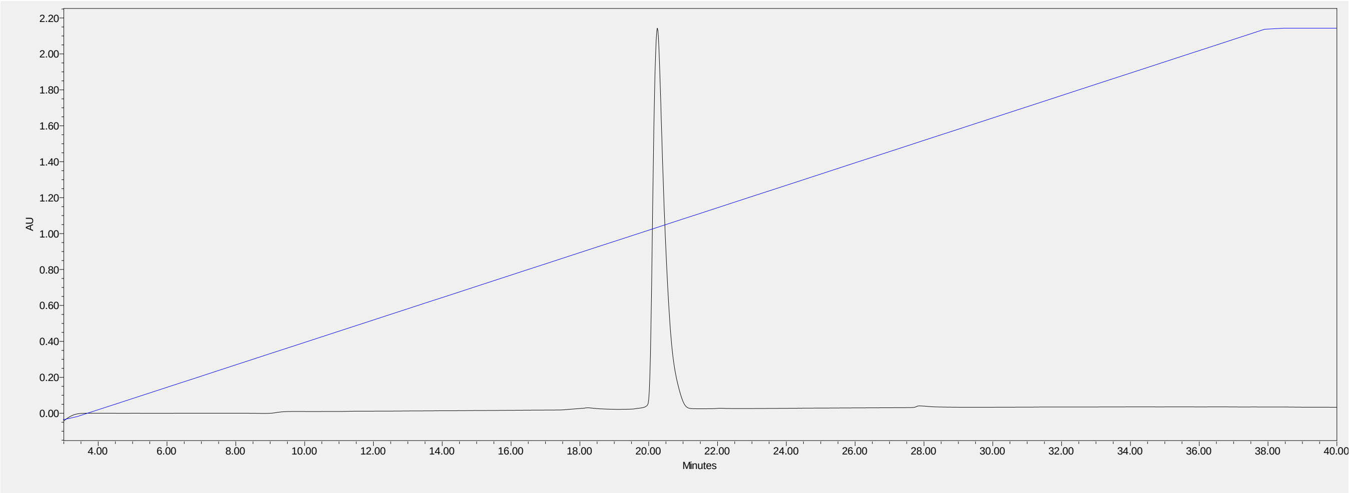
AU
Minutes
2.20
2.00
1.80
1.60
1.40
1.20
1.00
0.80
0.60
0.40
0.20
0.00
4.00
6.00
8.00
10.00
12.00
14.00
16.00
18.00
20.00
22.00
24.00
26.00
28.00
30.00
32.00
34.00
36.00
38.00
40.00

(C)

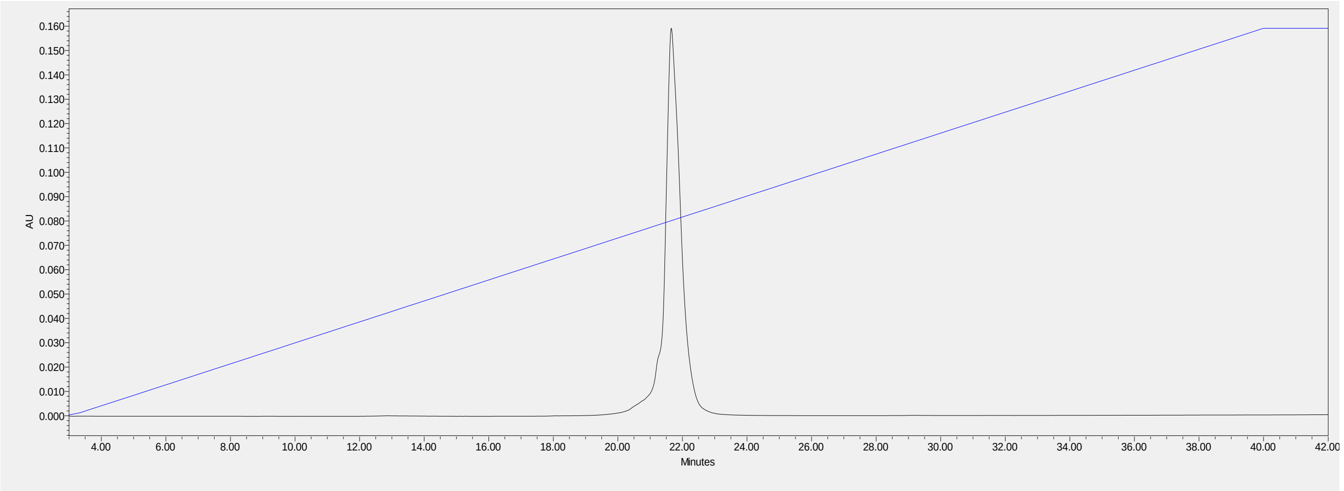
AU
Minutes
0.160
0.150
0.140
0.130
0.120
0.110
0.100
0.090
0.080
0.070
0.060
0.050
0.040
0.030
0.020
0.010
0.000
4.00
6.00
8.00
10.00
12.00
14.00
16.00
18.00
20.00
22.00
24.00
26.00
28.00
30.00
32.00
34.00
36.00
38.00
40.00
42.00

(D)

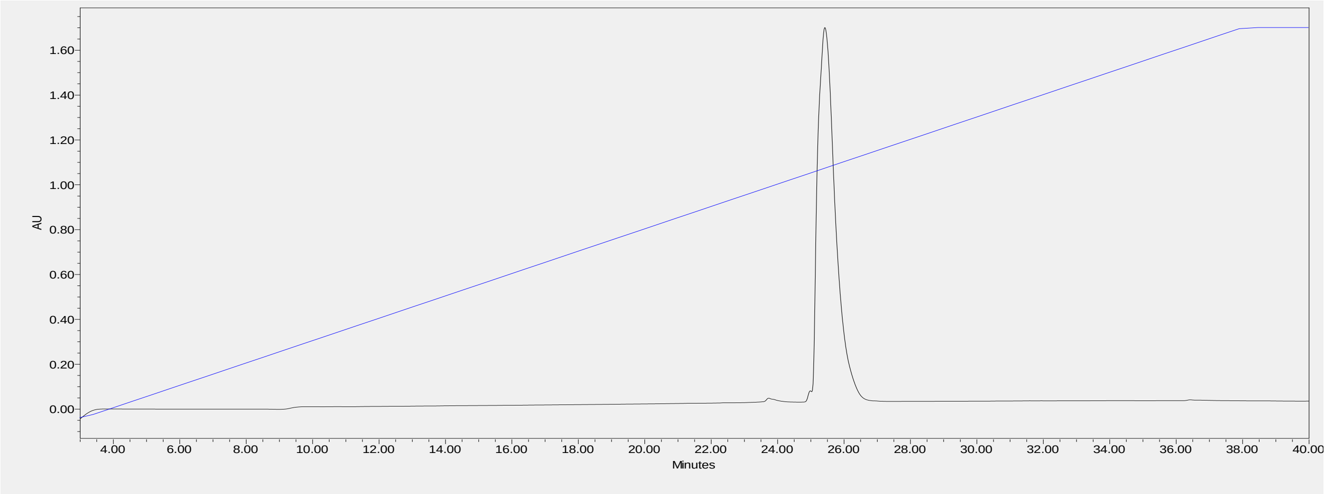

AU
1.60
1.40
1.20
1.00
0.80
0.60
0.40
0.20
0.00
4.00
6.00
8.00
10.00
12.00
14.00
16.00
18.00
20.00
22.00
24.00
26.00
28.00
30.00
32.00
34.00
36.00
38.00
40.00
Minutes

(E)

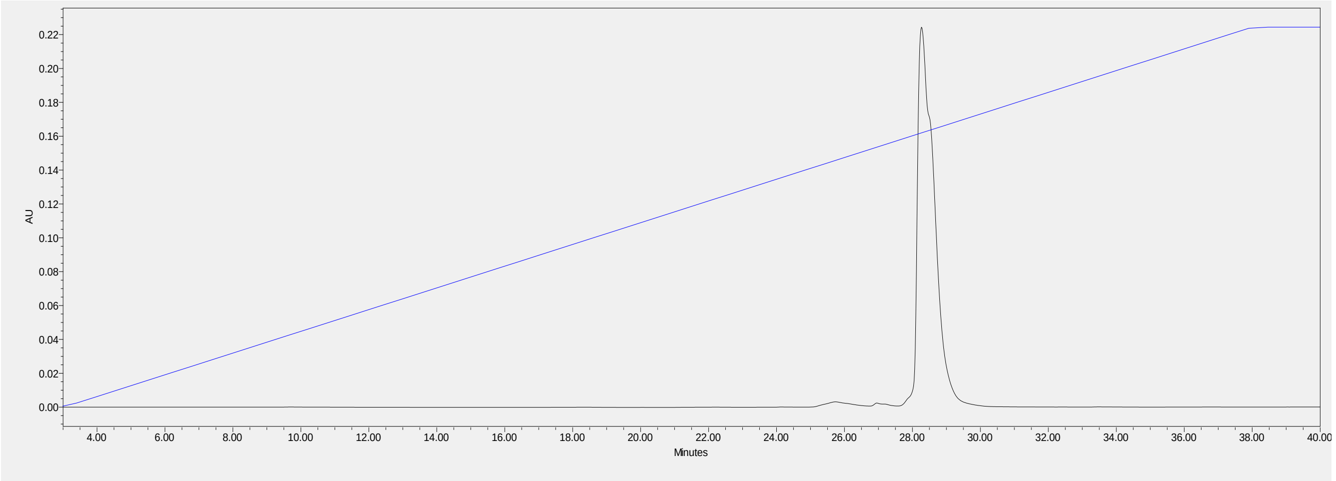

AU
0.22
0.20
0.18
0.16
0.14
0.12
0.10
0.08
0.06
0.04
0.02
0.00
4.00
6.00
8.00
10.00
12.00
14.00
16.00
18.00
20.00
22.00
24.00
26.00
28.00
30.00
32.00
34.00
36.00
38.00
40.00
Minutes

(F)

Figure S2. Reverse phase HPLC chromatogram of purified peptides (**A**) R2AW, (**B**) [Ser^{23,29}]R2AW, (**C**) R2AW(1-22), (**D**) R2AW(1-22)-NH_2, (**E**) [Lys^{4,19}, Leu^{20}]R2AW(1-22)-NH_2, and (**F**) [Trp^{6,10}]R2AW(1-22)-NH_2 monitored at 214 nm. The acetonitrile gradient is indicated by solid line. The peaks indicate the retention time of the pure fragment of the peptide.

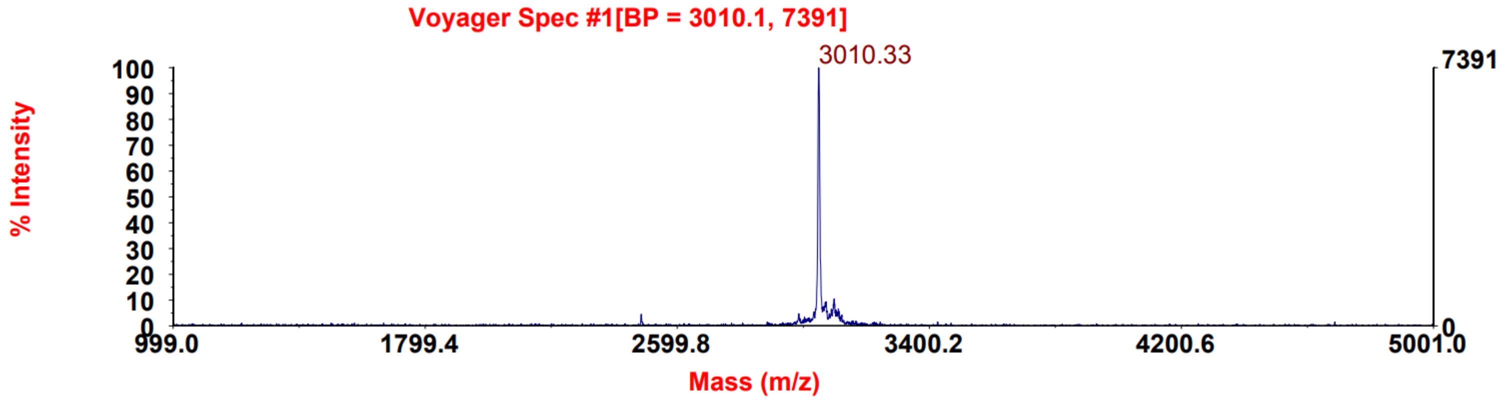

(A)

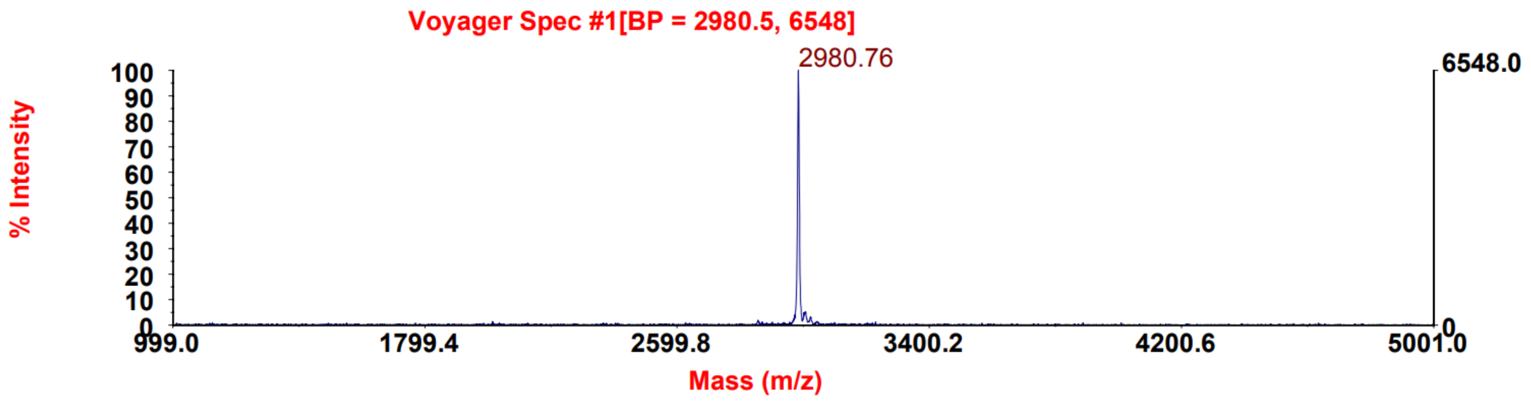
Voyager Spec #1[BP = 2980.5, 6548]
2980.76
6548.0
% Intensity
100
90
80
70
60
50
40
30
20
10
0
0
999.0
1799.4
2599.8
3400.2
4200.6
5001.0
Mass (m/z)

(B)

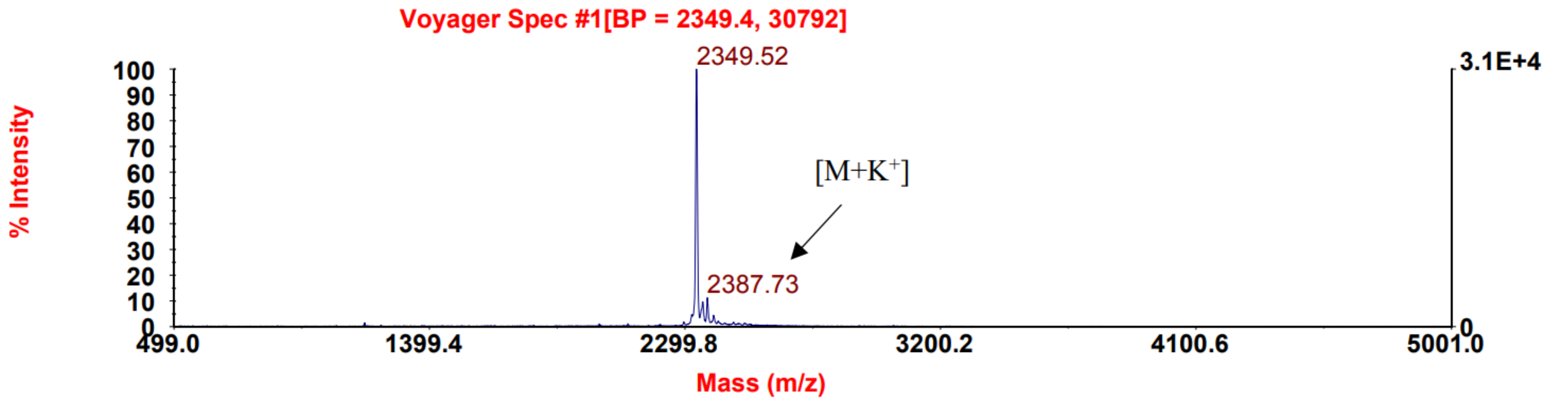
Voyager Spec #1[BP = 2349.4, 30792]
2349.52
3.1E+4
% Intensity
100
90
80
70
60
50
40
30
20
10
0
0
[M+K+]
2387.73
499.0
1399.4
2299.8
3200.2
4100.6
5001.0
Mass (m/z)

(C)

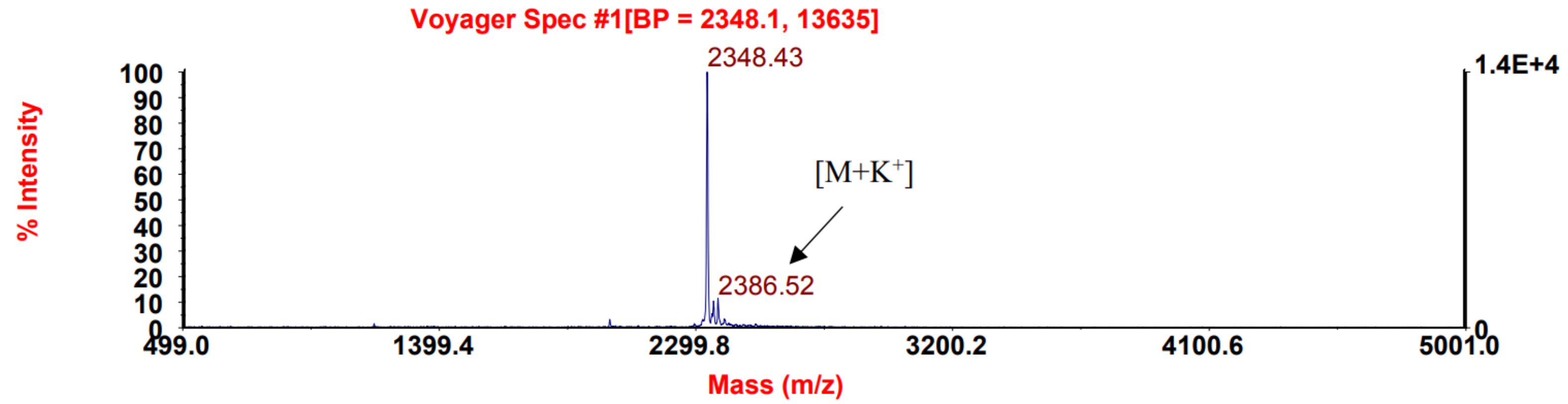
Voyager Spec #1[BP = 2348.1, 13635]
2348.43
1.4E+4
[M+K+]
2386.52
% Intensity
100
90
80
70
60
50
40
30
20
10
0
499.0
1399.4
2299.8
3200.2
4100.6
5001.0
0
Mass (m/z)

(D)

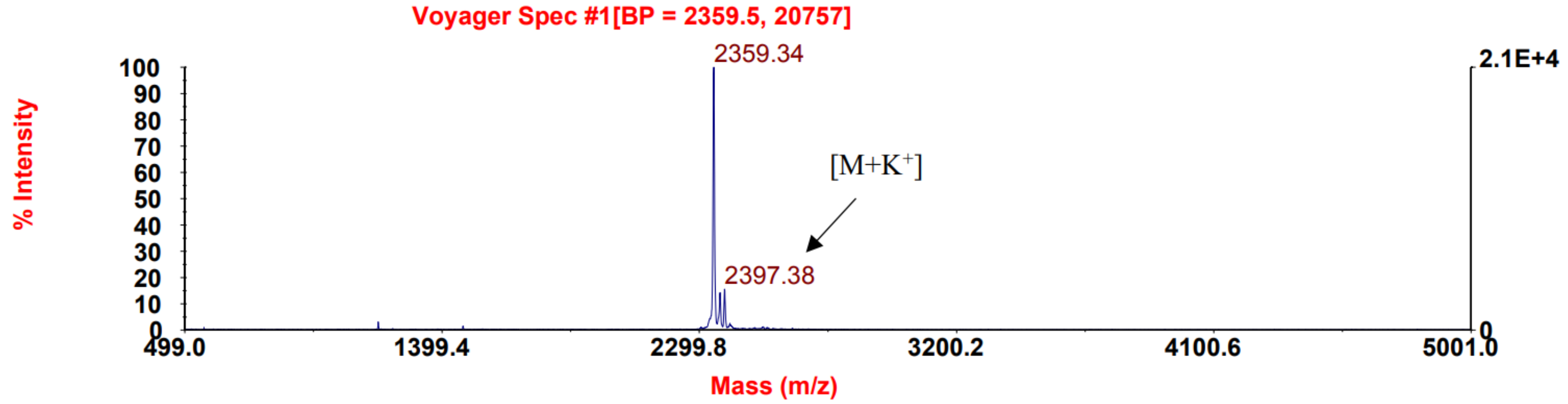
Voyager Spec #1[BP = 2359.5, 20757]
2359.34
2.1E+4
[M+K+]
2397.38
% Intensity
100
90
80
70
60
50
40
30
20
10
0
499.0
1399.4
2299.8
3200.2
4100.6
5001.0
0
Mass (m/z)

(E)

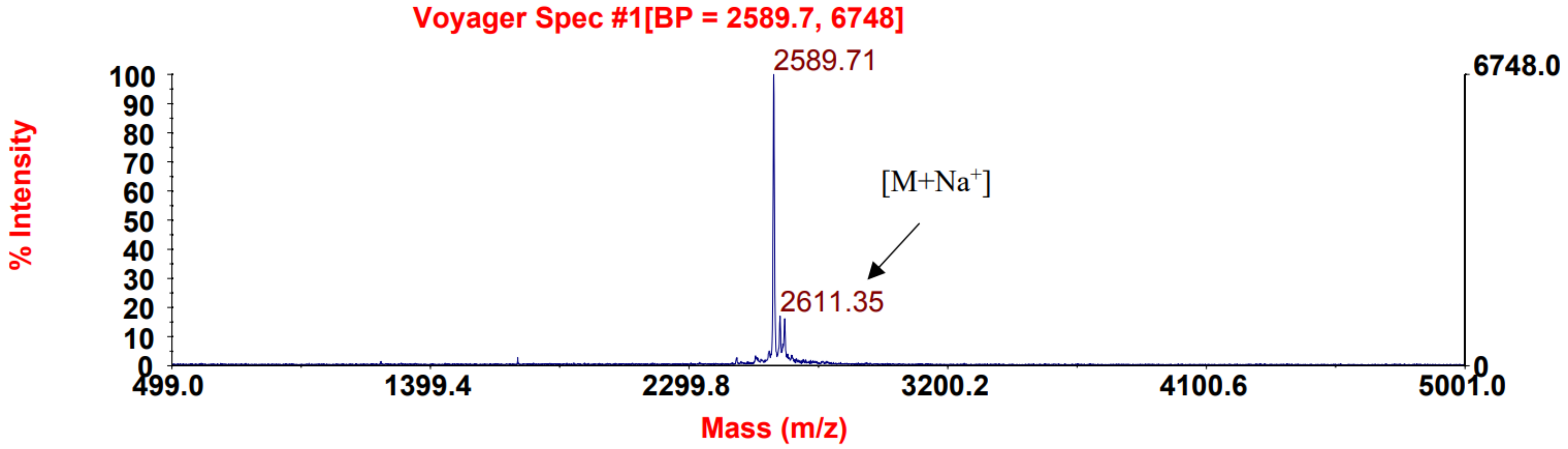

(F)

Figure S3. MALDI-TOF mass spectra of (**A**) R2AW, (**B**) [Ser[23,29]]R2AW, (**C**) R2AW(1-22), (**D**) R2AW(1-22)-NH_2, (**E**) [Lys[4,19], Leu[20]]R2AW(1-22)-NH_2, and (**F**) [Trp[6,10]]R2AW(1-22)-NH_2. The abscissa represents the region of mass-to-charge ratio (m/z). The ordinate indicates the percentage of signal intensity. BP means base peak.
